# Supplementary material for: QSTR Modeling to Find Relevant DFT Descriptors Related to the Toxicity of Carbamates
Source: Molecules. 2022 Aug 28;27(17):5530. doi: 10.3390/molecules27175530 (PMC9457808; doi:10.3390/molecules27175530)
Supplement: Supplementary file 1 [file molecules-27-05530-s001.zip › OtherMethods.pdf]

In the first step, we report the variables with a high value of correlation.

X	Y	correlation
Mulliken_electronegativity.. eV.	Chemical_potential..eV.	-1
Mu..	Electrophilicity_index..eV.	0.987
Hardness..eV.	Softness..eV..1.	-0.975
Electrophilicity_index..eV.	w.	0.968
Vertical_EA..eV.	Mu...1	0.967

The following results were performed using the Full data file from supplementary material, we randomly selected training and test data subsets to validate different regression models. In all cases, we reported the coefficients of the linear regression model for each variable. Moreover, the R-squared, Adjusted R-squared, and the mean square error (mse) for training and test data values. All tables report the significant level as a measure of its p-value variation through the symbols such as *0 '***' 0.001 '**' 0.01 '*' 0.05 '.' 0.1 ' ' 1.*

Quadratic Least Square Regression

Table S1 Coefficients for quadratic least Squared regression model

	Estimate	***Std. Error***	***t value***	***Pr(>|t|)***
(Intercept)	*1.275e+01*	*9.521e+00*	*1.340*	*0.183283*
Volume..Ang..3.	*5.122e-03*	*5.352e-03*	*0.957*	*0.340758*
W..g.mol	*4.795e-03*	*5.798e-03*	*-0.827*	*0.410139*
Vertical_IP..eV.	*2.052e+03*	*1.007e+03*	*2.038*	*0.044109 **
Vertical_EA..eV.	*-1.587e+03*	*1.011e+03*	*-1.570*	*0.119367*
Mulliken_electronegativity..eV.	*-7.287e+01*	*1.328e+03*	*-0.055*	*0.956329*
Chemical_potential..eV.	*NA*	*NA*	*NA*	*NA*
Mu..	*NA*	*NA*	*NA*	*NA*
Mu...1	*NA*	*NA*	*NA*	*NA*
Hardness..eV.	*-1.770e+03*	*7.874e+02*	*-2.248*	*0.026707 **
Softness..eV..1.	*3.139e+01*	*3.499e+01*	*0.897*	*0.371814*
Electrophilicity_index..eV	*1.560e+03*	*1.582e+03*	*0.986*	*0.326345*
w.	*-7.775e+02*	*7.910e+02*	*-0.983*	*0.327953*
w..1	*NA*	*NA*	*NA*	*NA*
Nucleophilicity_index.eV.	*2.905e-01*	*2.643e-01*	*1.099*	*0.274181*
C_q.N.	*-2.700e+01*	*1.325e+01*	*-2.038*	*0.044086 **
D.LOC	*-5.743e-01*	*1.452e-01*	*-3.956*	*0.000139 ****
D.SpPosA_RG	*-3.815e+01*	*9.174e+00*	*-4.158*	*6.61e-05 ****
D.H4m	*-1.571e+00*	*6.911e-01*	*-2.273*	*0.025110 **
D.nCt	*2.049e-01*	*8.778e-02*	*2.334*	*0.021520 **
D.nROCON	*-7.479e-01*	*1.687e-01*	*-4.433*	*2.31e-05 ****
D.B05.C.N.	*8.326e-01*	*2.347e-01*	*3.548*	*0.000585 ****
D.B05.N.O.	*4.051e-01*	*1.222e-01*	*3.314*	*0.001264 ***
D.DLS_05	*7.227e-01*	*1.671e-01*	*4.325*	*3.50e-05 ****

Residual standard error: 0.4616 on 104 degrees of freedom

Multiple R-squared: 0.721, ***Adjusted R-squared: 0.67***

F-statistic: 14.14 on 19 and 104 DF, p-value: < 2.2e-16

Training Error (mse): 0.178698

Test error (mse): 0.389001

Stepwise Selection (backward-forward)

Table S2 Coefficients for stepwise selection regression model

	Estimate	*Std. Error*	*t value*	*Pr(>|t|)*
(Intercept)	2.054e+01	3.500e+00	5.869	4.71e-08 ***
Vertical_IP..eV.	1.954e+03	7.504e+02	2.604	0.010480 *
Vertical_EA..eV.	-1.950e+03	7.504e+02	-2.599	0.010626 *
Hardness..eV.	-1.952e+03	7.504e+02	-2.601	0.010581 *
Electrophilicity_index..eV	6.548e+00	2.073e+00	3.158	0.002051 **
C_q.N.	-3.264e+01	1.227e+01	-2.660	0.008985 **
D.LOC	-5.902e-01	1.205e-01	-4.897	3.36e-06 ***
D.SpPosA_RG	-2.860e+01	5.433e+00	-5.264	7.04e-07 ***
D.H4m	-1.634e+00	3.957e-01	-4.129	7.12e-05 ***
D.nCt	1.679e-01	8.205e-02	2.046	0.043101 *
D.nROCON	-7.566e-01	1.590e-01	-4.757	6.01e-06 ***
D.B05.C.N.	7.439e-01	2.224e-01	3.345	0.001125 **
D.B05.N.O.	3.820e-01	1.125e-01	3.396	0.000951 ***
D.DLS_05	7.500e-01	1.504e-01	4.987	2.31e-06 ***

Residual standard error: 0.4571 on 110 degrees of freedom

Multiple R-squared: 0.7106, **Adjusted R-squared: 0.6764**

F-statistic: 20.78 on 13 and 110 DF, p-value: < 2.2e-16

This model has 13 prediction variables.

Training Error (mse): 0.185328

Test error (mse): 0.331688

Ridge and Lasso Regression

In order to avoid predictor multicollinearity and overfitting problems. We improved the value of Test error using Ridge and Lasso regularization methods. We improve the values of the mean square error for test data in both cases. Ridge method found 14 coefficients with values greater than 0.1 while Lasso obtains 12. Both methods agree on the most relevant variables in the linear regression model were *Softness..eV..1., C_q.N., D.SpPosA_RG.*

Table S3 Coefficients for Ridge and Lasso regularization techniques in regression model

	Ridge	***Lasso***
(Intercept)	14.63259	14.1059705
Volume..Ang..3.	-0.00040194	0.0
W..g.mol	-0.00037490	0.0
Vertical_IP..eV.	0.05665028	*0.0*
Vertical_EA..eV.	*0.00623561*	*0.0*
Mulliken_electronegativity..eV.	0.1529933	*0.0*
Chemical_potential..eV.	-0.01500953	*0.0*
Mu..	-0.01230700	*0.0*
Mu...1	-0.09916744	-0.1646949
Hardness..eV.	-0.01772124	*0.0*
Softness..eV..1.	5.008295	4.7593784
Electrophilicity_index..eV	0.1204536	*0.0*
w.	0.3398070	*0.0*
w..1	-0.00918989	*0.0*
Nucleophilicity_index.eV.	0.1065011	0.1617319
C_q.N.	-28.95033	-32.2040285
D.LOC	-0.4892636	-0.5594187
D.SpPosA_RG	-18.14742	-19.2866898
D.H4m	-1.057055	-1.2929849
D.nCt	0.1682489	0.1377855
D.nROCON	-0.5254125	-0.6538064
D.B05.C.N.	0.5608538	0.5641765
D.B05.N.O.	0.2355432	0.2870240
D.DLS_05	0.6136216	0.7175967
Training Error (MSE)	**0.225995**	**0.220621**
Test Error (MSE)	**0.249440**	**0.242499**
R-squared (cross validation)	**0.6674146**	**0.6669366**

Moreover, the Extreme Gradient Boosting and Support Vector Regression (SVR) regression models using xgboost and sklearn.svr libraries in the python language were realized. The hyperparameters used can be visualized in the supplementary code. The prediction accuracy and the mean squared error are displaying in the next table:

	**XGBoost**	**SVR**
**Score ()**	0.768177	0.889720
**RMSE**	0.369744	0.255018

Similar results to Ridge and Lasso regularization techniques were obtained. XGBoost and SVR don’t provide an explicit multilinear equation, unlike a classical multilinear model.
