# Supplementary material for: QSTR Modeling to Find Relevant DFT Descriptors Related to the Toxicity of Carbamates
Source: Molecules. 2022 Aug 28;27(17):5530. doi: 10.3390/molecules27175530 (PMC9457808; doi:10.3390/molecules27175530)
Supplement: Supplementary file 1 [file molecules-27-05530-s001.zip › Supplementary Materials.pdf]

Supplementary Materials

QSTR modeling to find relevant DFT descriptors related to the toxicity of carbamates

Emma H. Acosta-Jiménez[1], Luis A. Zárate-Hernández[1], Rosa L. Camacho-Mendoza[1], Simplicio González-Montiel[1], José G. Alvarado-Rodríguez[1], Carlos Z. Gómez-Castro[1], Miriam Pescador-Rojas[2], Amilcar Meneses-Viveros[3] and Julián Cruz-Borbolla[1,*]

[1] Área Académica de Química, Centro de Investigaciones Químicas, Universidad Autónoma del Estado de Hidalgo, km. 4.5 Carretera Pachuca-Tulancingo, Ciudad del Conocimiento, C.P. 42184, Mineral de la Reforma, Hidalgo, México

[2] Escuela Superior de Cómputo, Instituto Politécnico Nacional, México

[3] Departamento de Computación, CINVESTAV-IPN, Av. IPN 2508, Col. San Pedro Zacatenco, Ciudad de México 07360, México

.

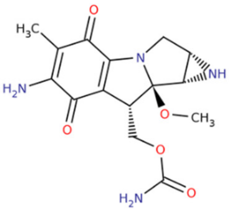
0000050077

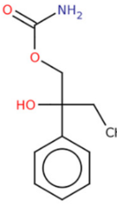
0000050191

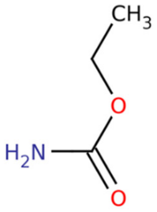
0000051796

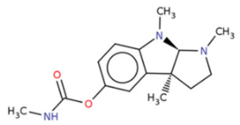
0000057476

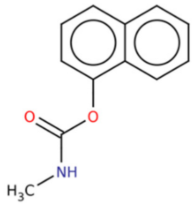
0000063252

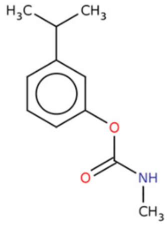
0000064006

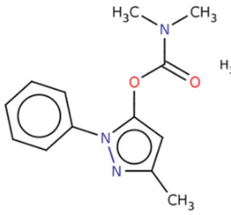
0000087478

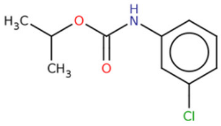
0000101213

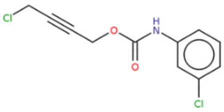
0000101279

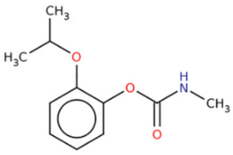
0000114261

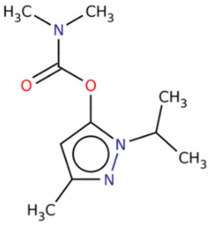
0000119380

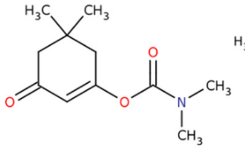
0000122156

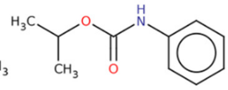
0000122429

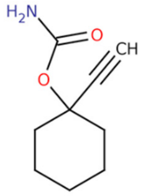
0000126523

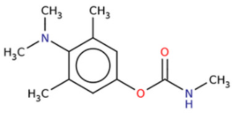
0000315184

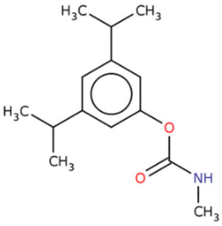
0000330643

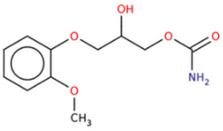
0000532036

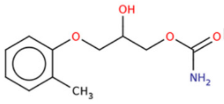
0000533062

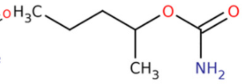
0000541957

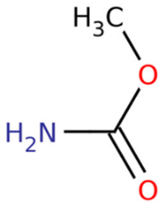
0000598550

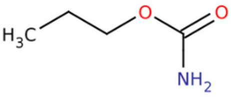
0000627123

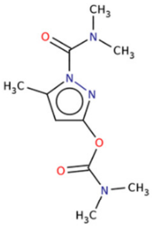
0000644644

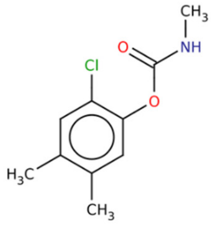
0000671045

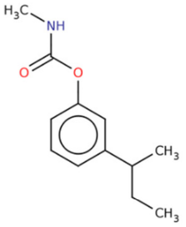
0000673198

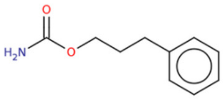
0000673314

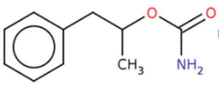
0000709900

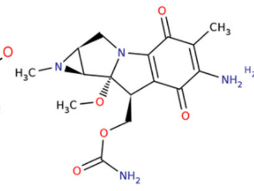
0000801525

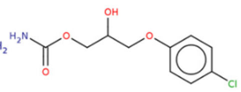
0000886748

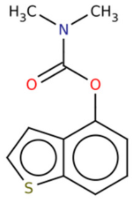
0001016462

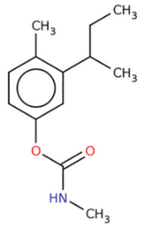
0001020355

Figure S1. Bidimensional structure of full carbamates set.

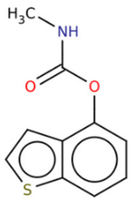
0001079330

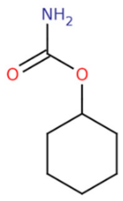
0001124545

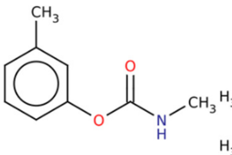
0001129415

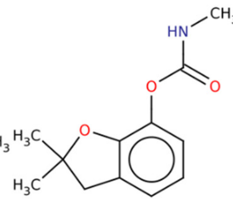
0001563662

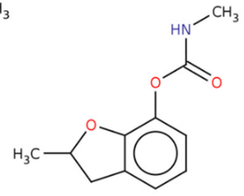
0001563673

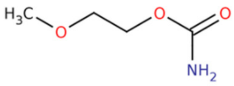
0001616882

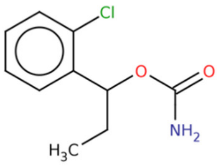
0001690331

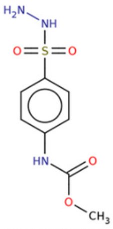
0001879261

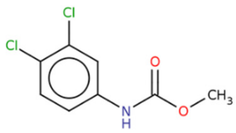
0001918189

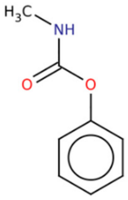
0001943799

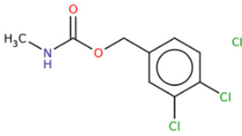
0001966581

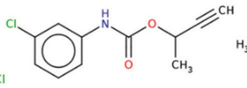
0001967164

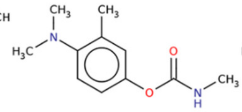
0002032599

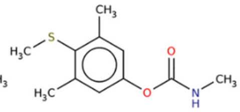
0002032657

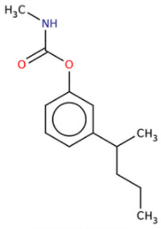
0002282340

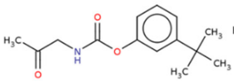
0002313919

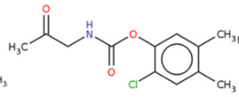
0002313931

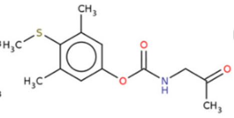
0002410095

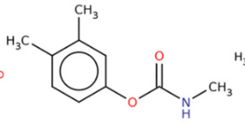
0002425107

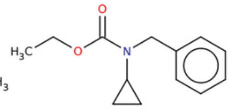
0002521019

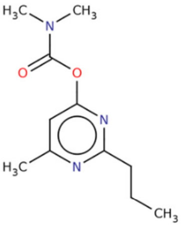
0002532492

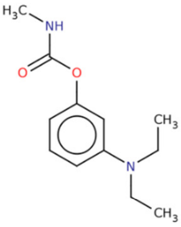
0002631267

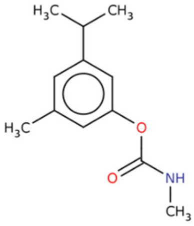
0002631370

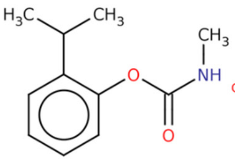
0002631405

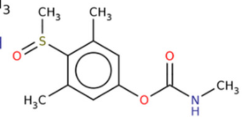
0002635101

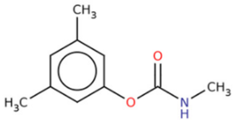
0002655143

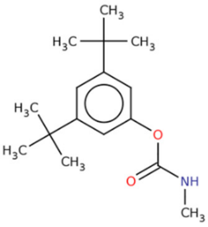
0002655198

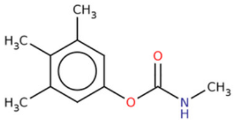
0002686999

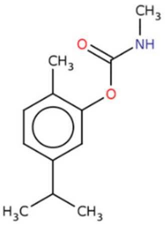
0002752718

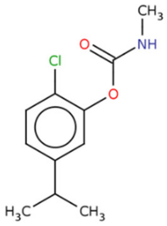
0002752810

Figure S1. (Cont.)

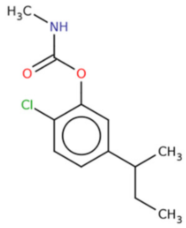
0002917193

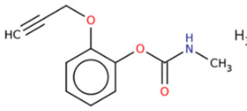
0003279467

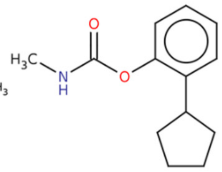
0003282006

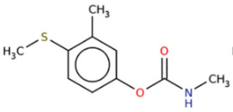
0003566005

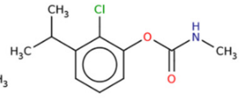
0003566094

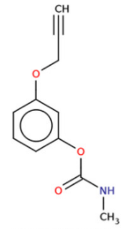
0003692908

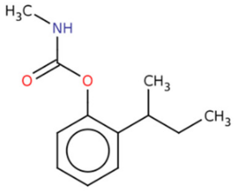
0003766812

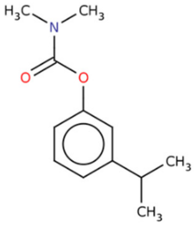
0003938452

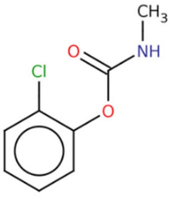
0003942549

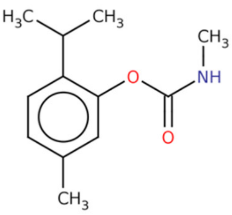
0003942710

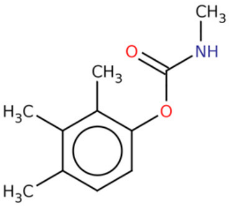
0003971899

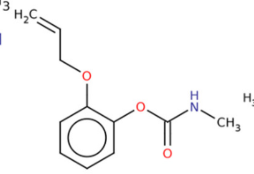
0004062991

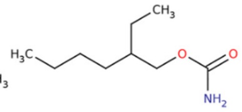
0004248219

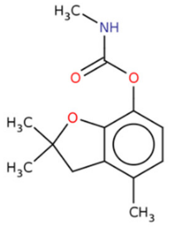
0004785722

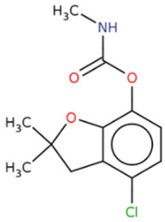
0004790925

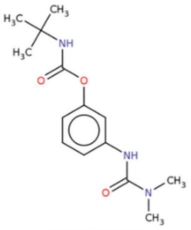
0004849325

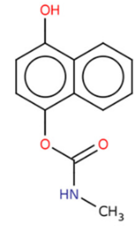
0005266977

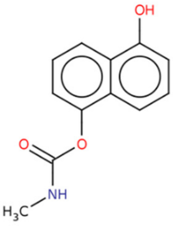
0005721722

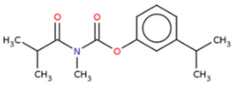
0005748265

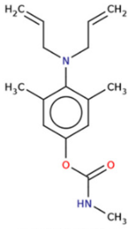
0006392467

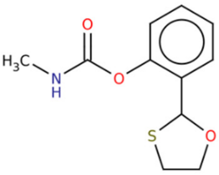
0006842348

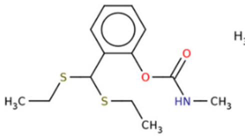
0006853798

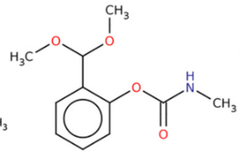
0006884599

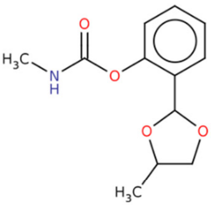
0006988201

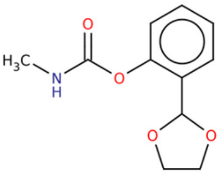
0006988212

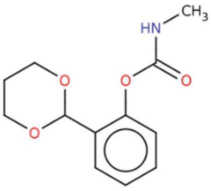
0007070635

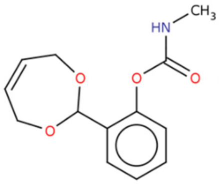
0007070715

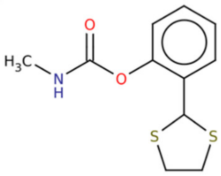
0007070931

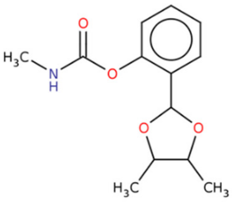
0007122045

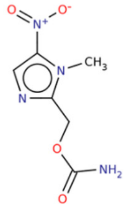
0007681767

Figure S1. (Cont.)

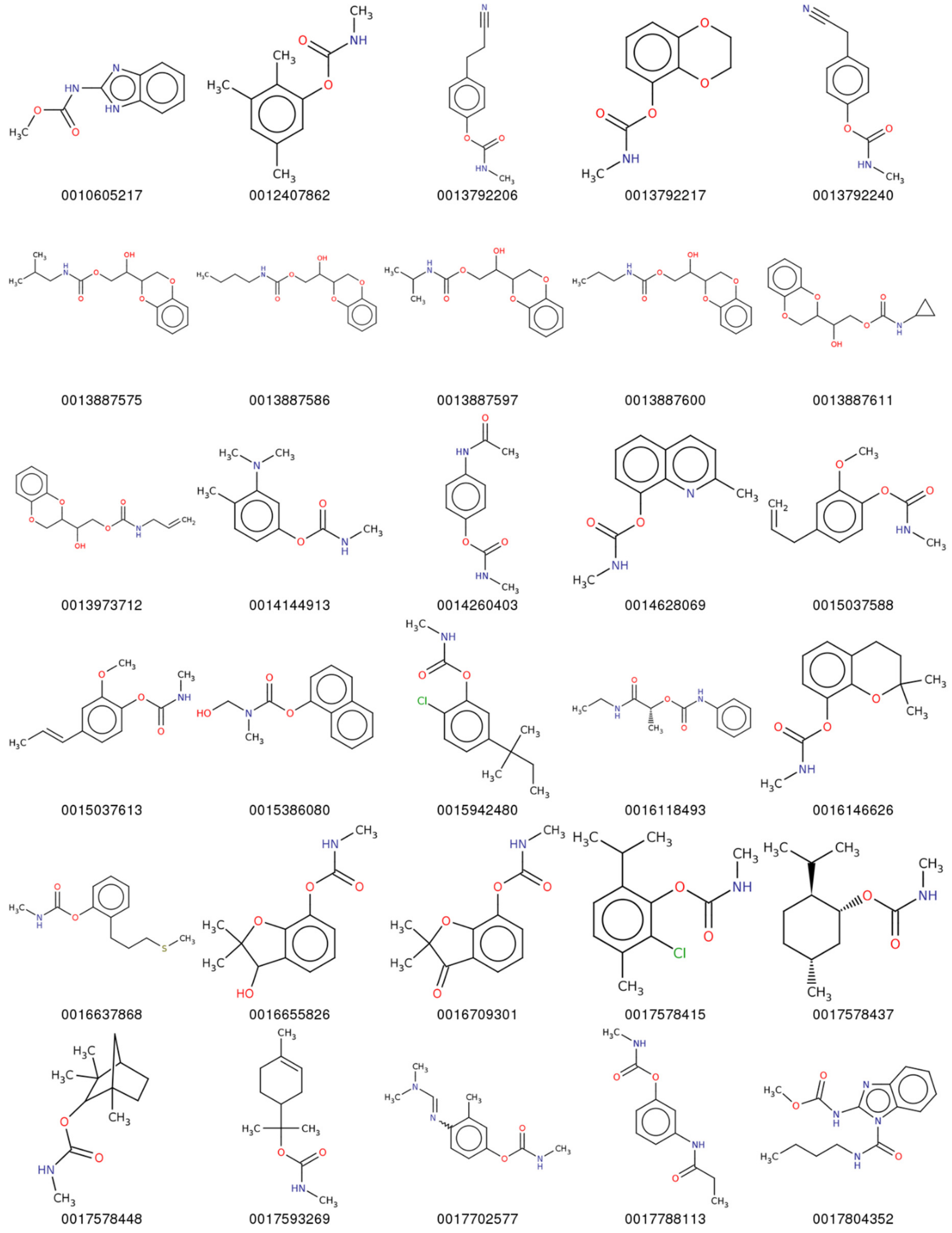

Figure S1. (Cont.)

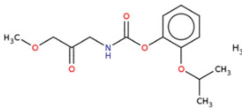
0017959114
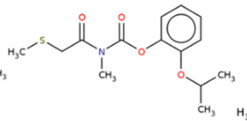
0017959125
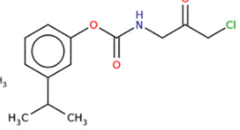
0017959330
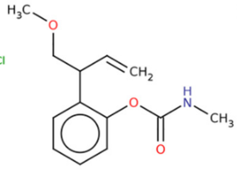
0018188175
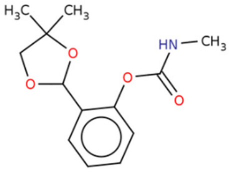
0018493215
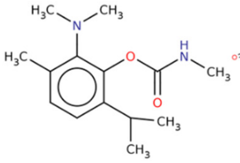
0018659455
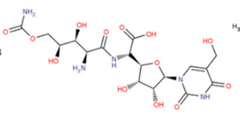
0019396066
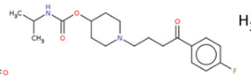
0020977508
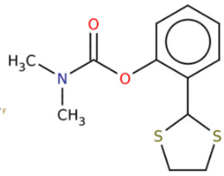
0021709444
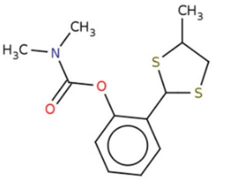
0022212937
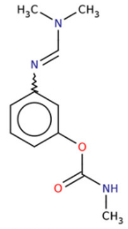
0022259309
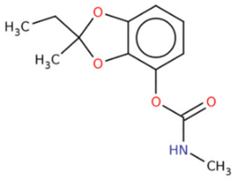
0022781244
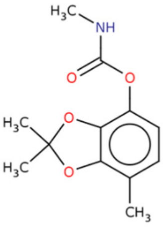
0022791204
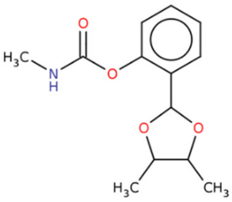
0022916014
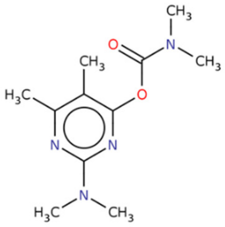
0023103982
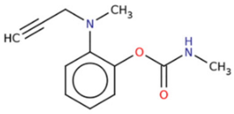
0023504076
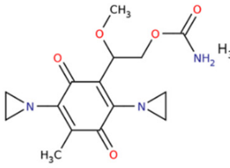
0024279912
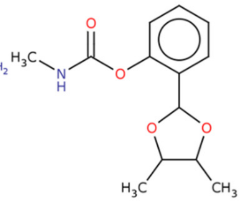
0024380856
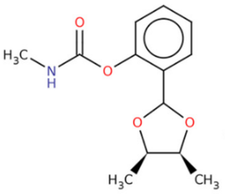
0024380867
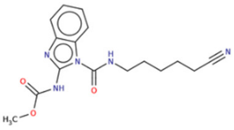
0028559004
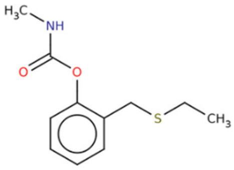
0029973135
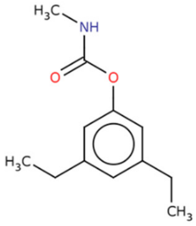
0030087479
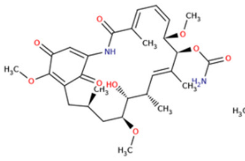
0030562346
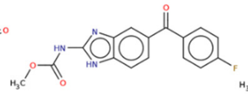
0031430156
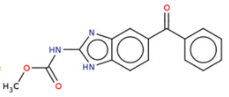
0031431397
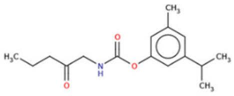
0034264249
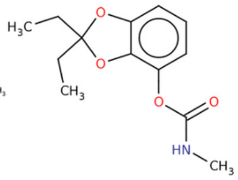
0040373317
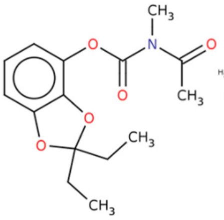
0040374149
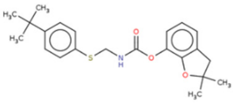
0050539703
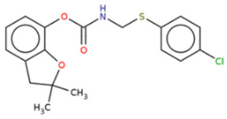
0050539736

Figure S1. (Cont.)

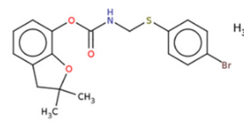
0050539747

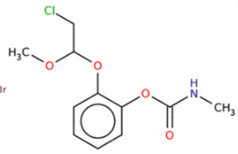
0051487695

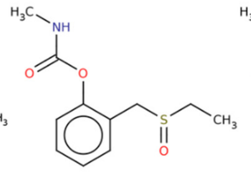
0053380226

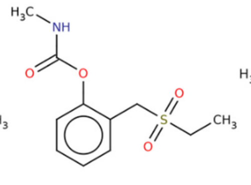
0053380237

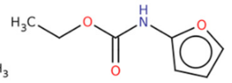
0054915627

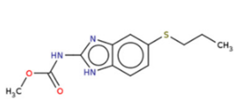
0054965218

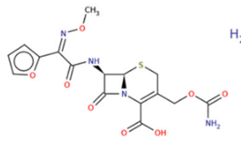
0055268752

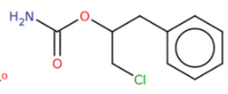
0060634631

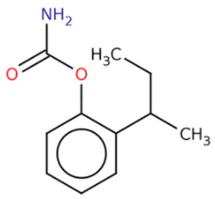
0061005127

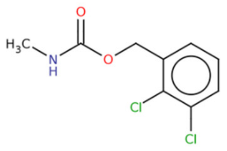
0062046371

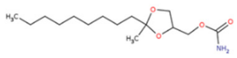
0063917486

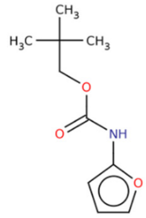
0063956901

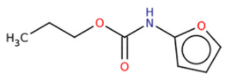
0063956912

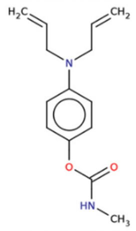
0064059149

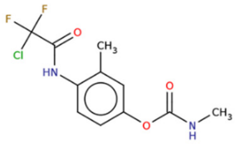
0064748158

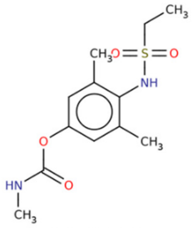
0064748216

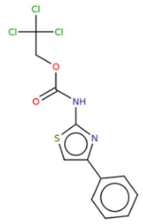
0071119103

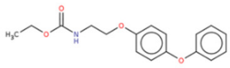
0072490018

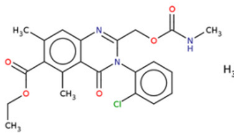
0082163551

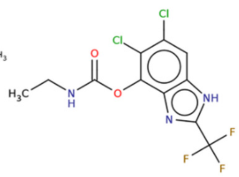
0089427190

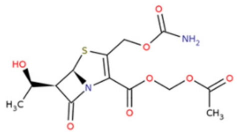
0099376224

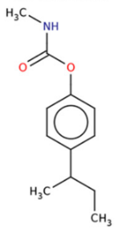
0100789693

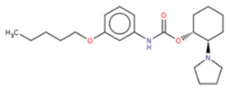
0104485010

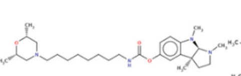
0154619519

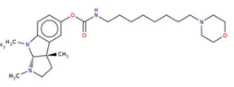
0154619564

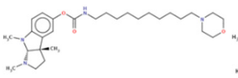
0154619644

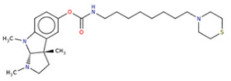
0154619666

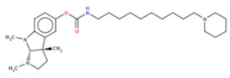
0154619917

Figure S1. (Cont.)

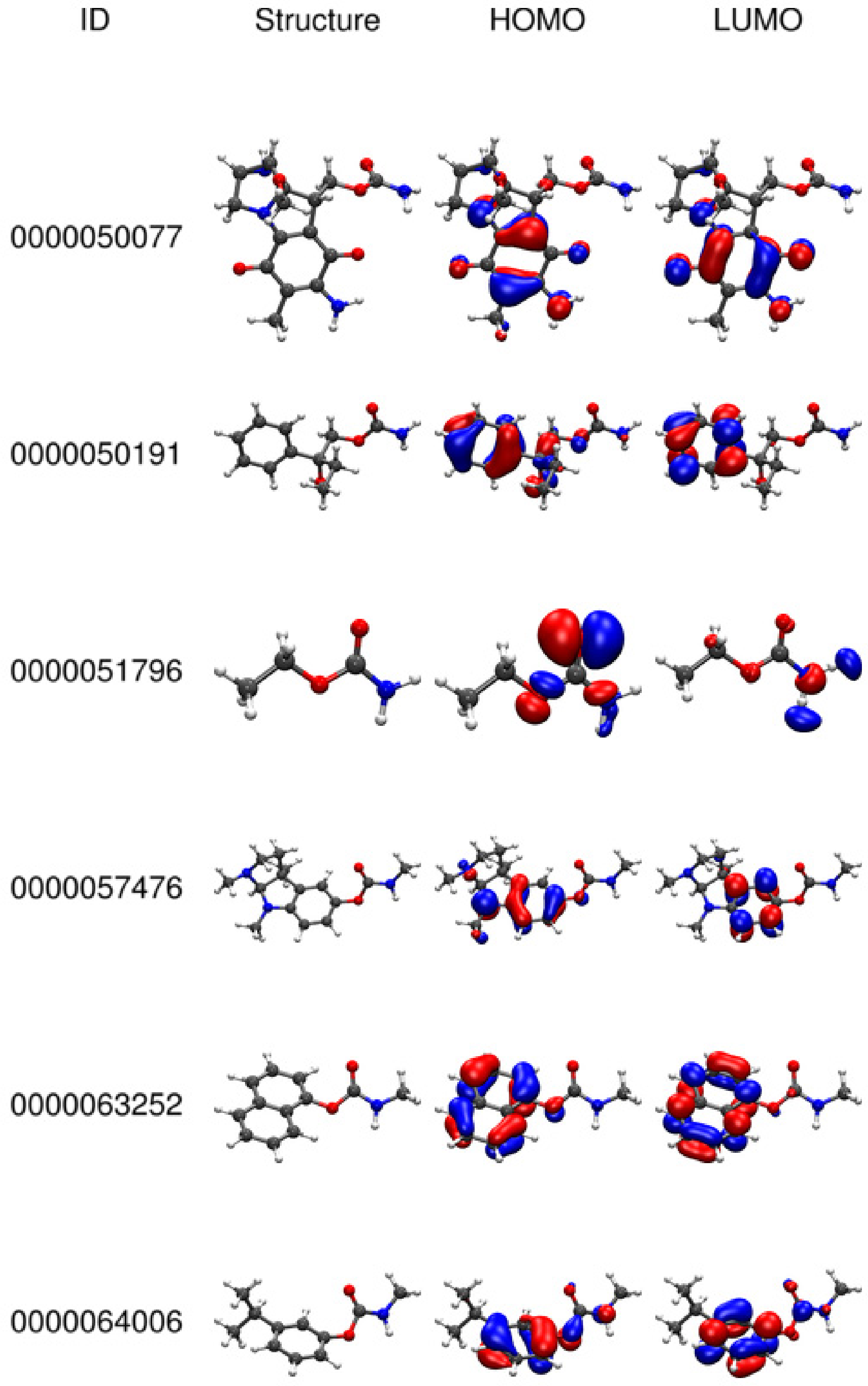

Figure S2. Tridimensional structure, HOMO and LUMO graphs of full carbamates set.

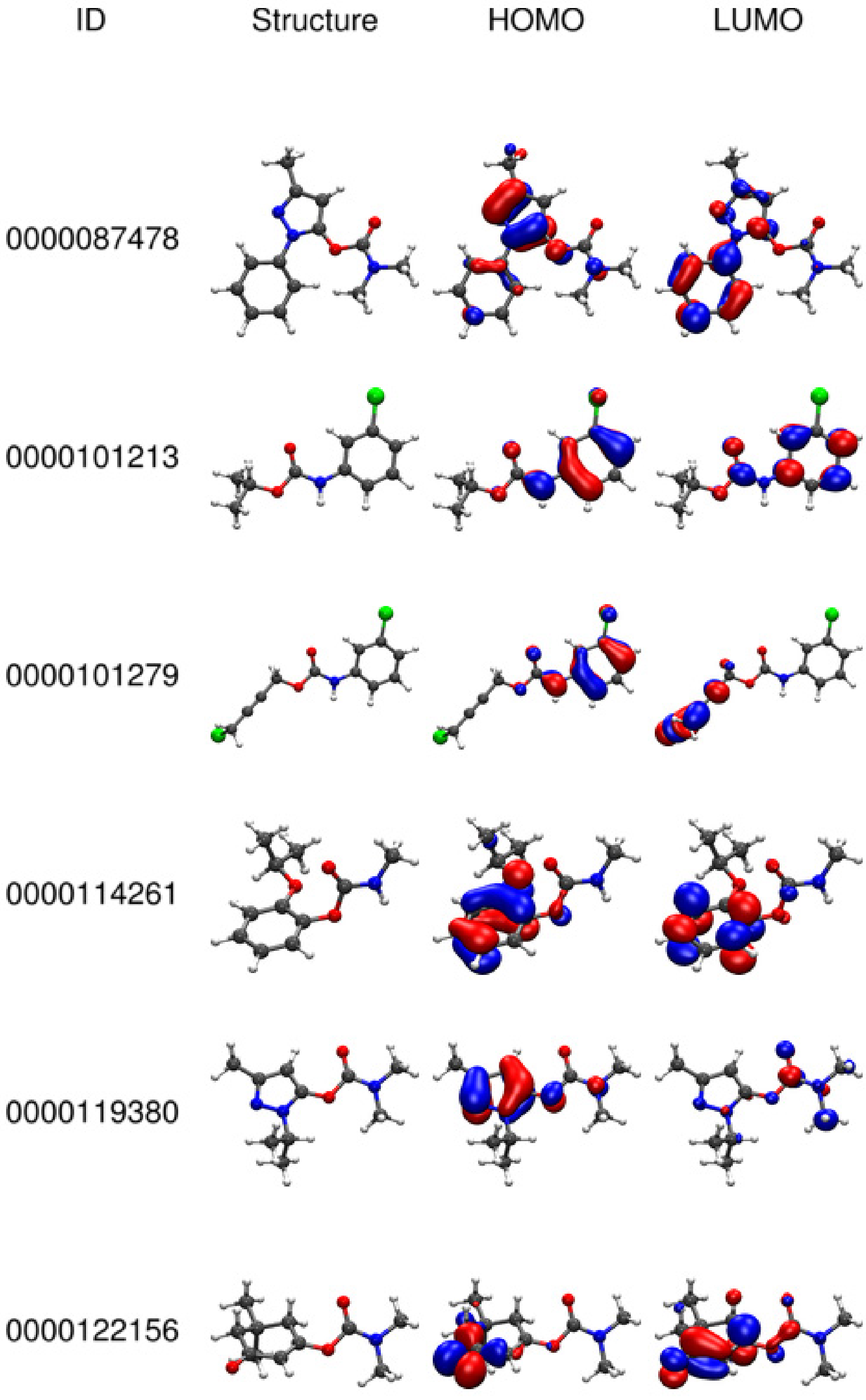

Figure S2. (Cont.)

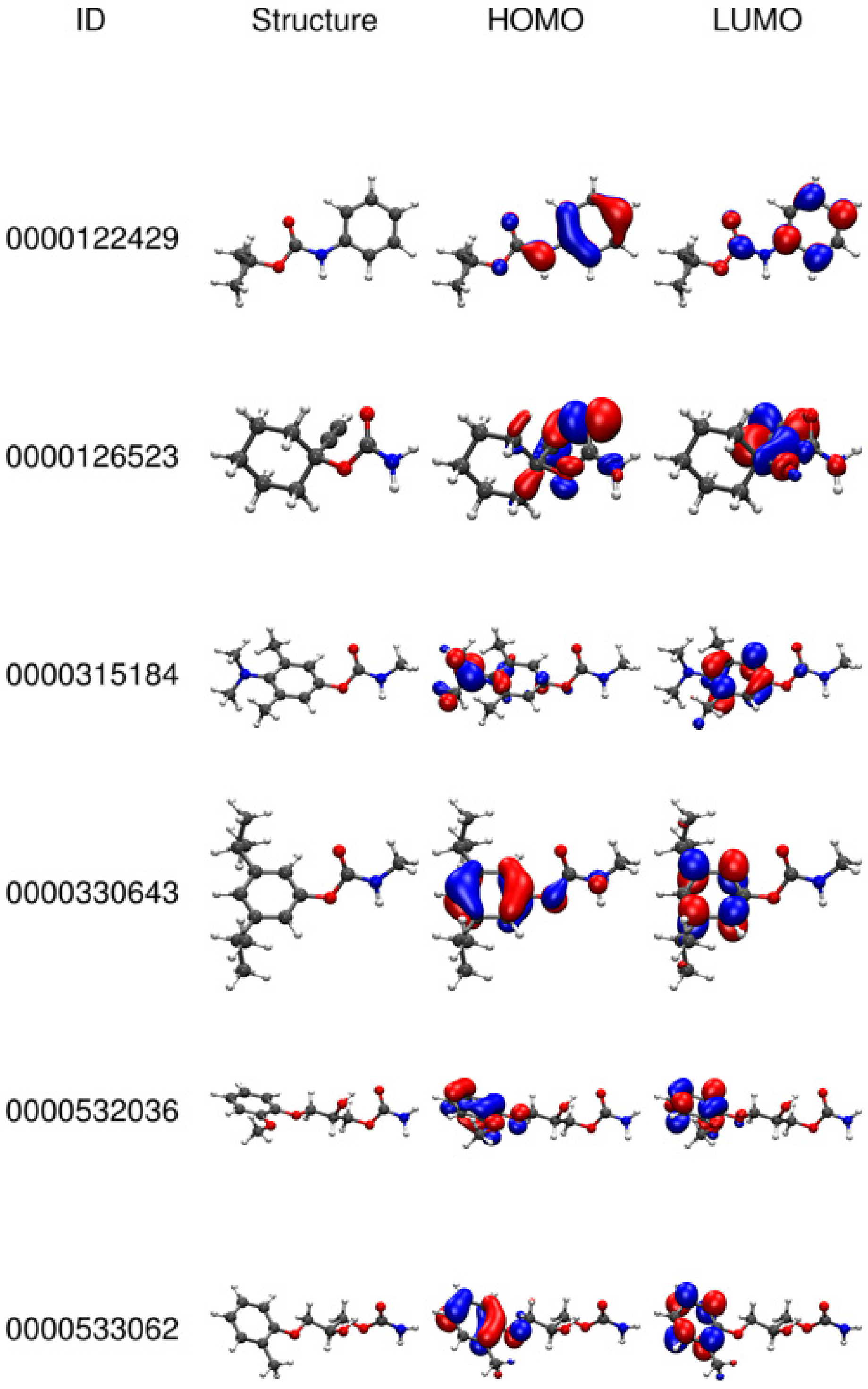

Figure S2. (Cont.)

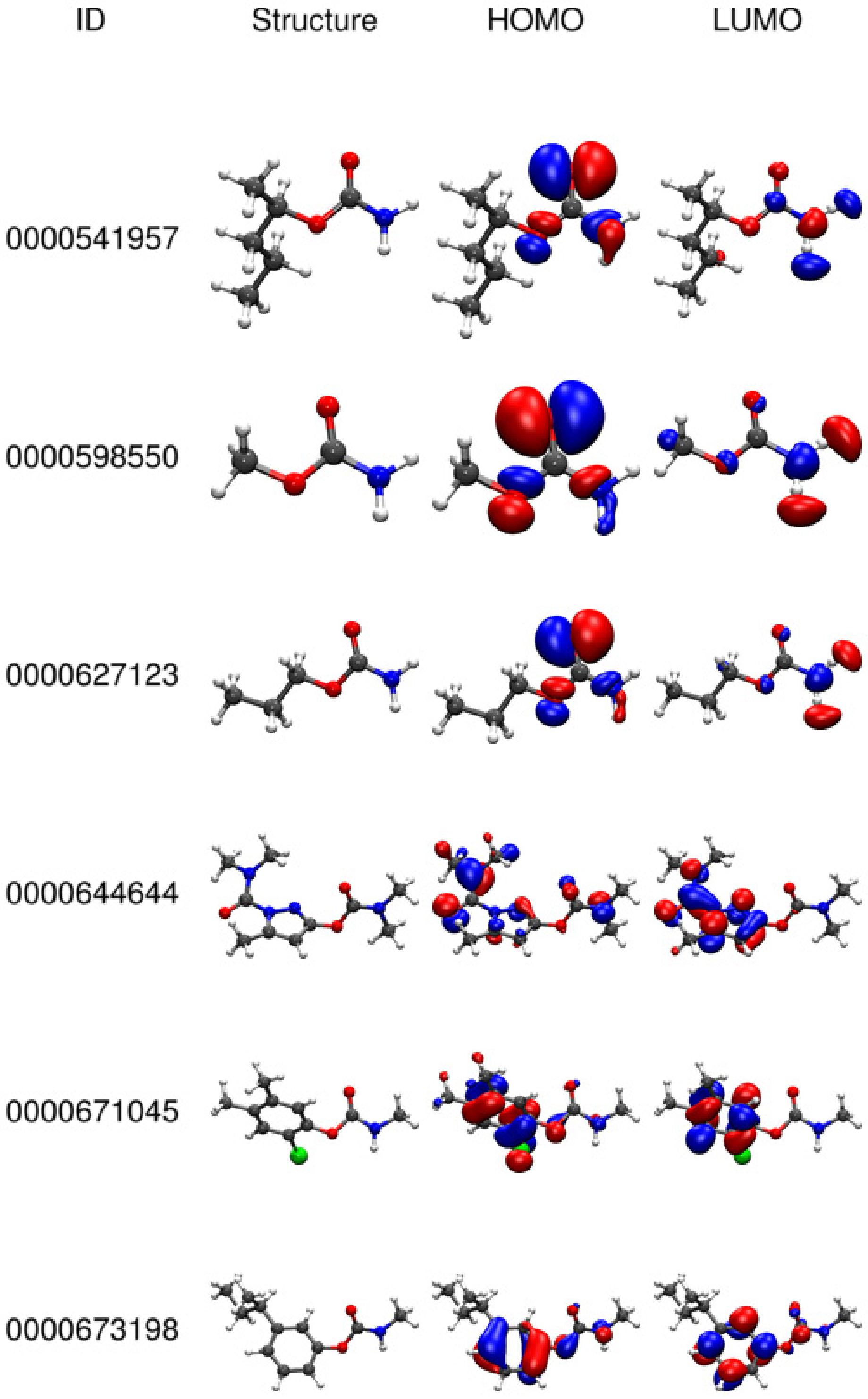

Figure S2. (Cont.)

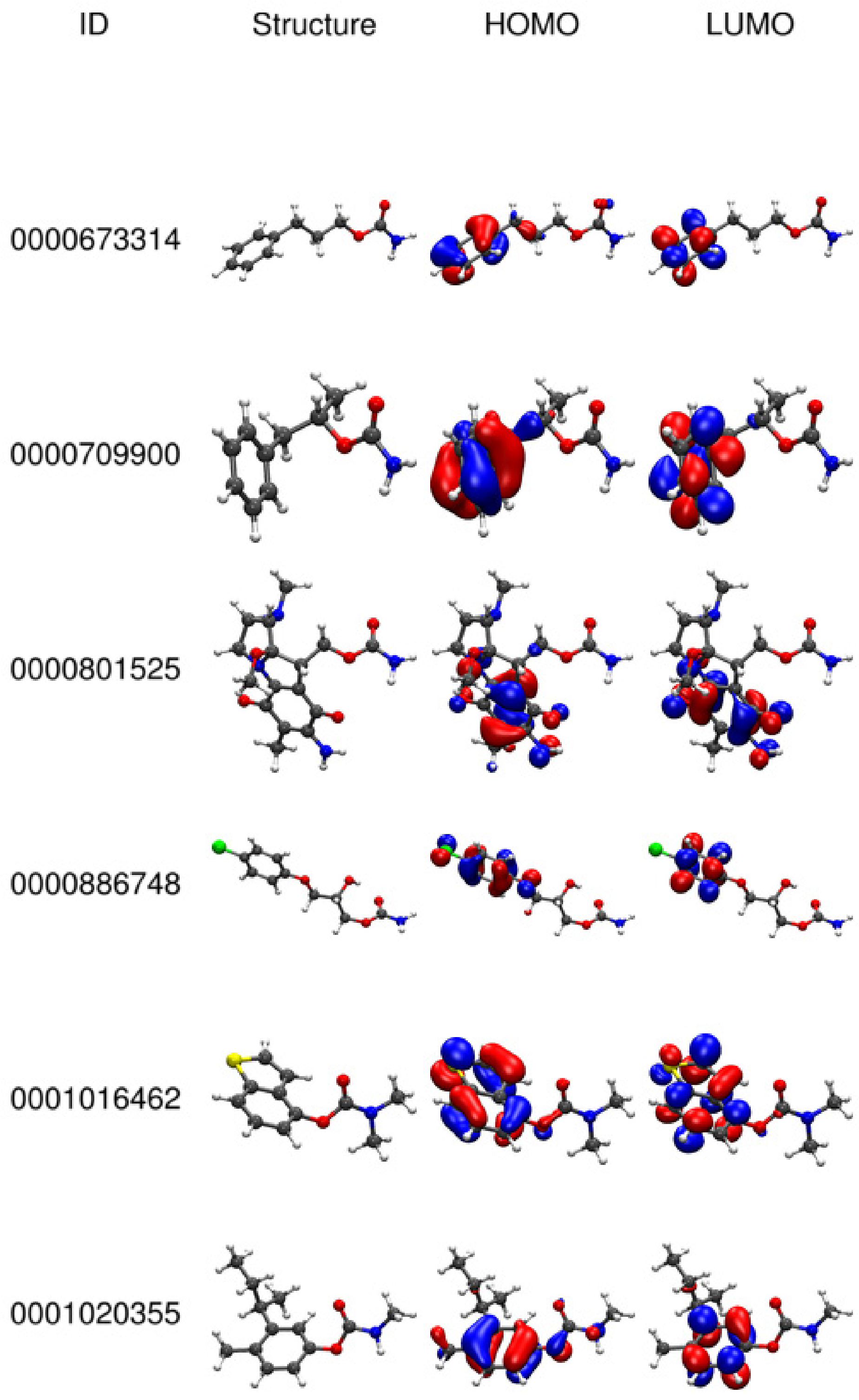

Figure S2. (Cont.)

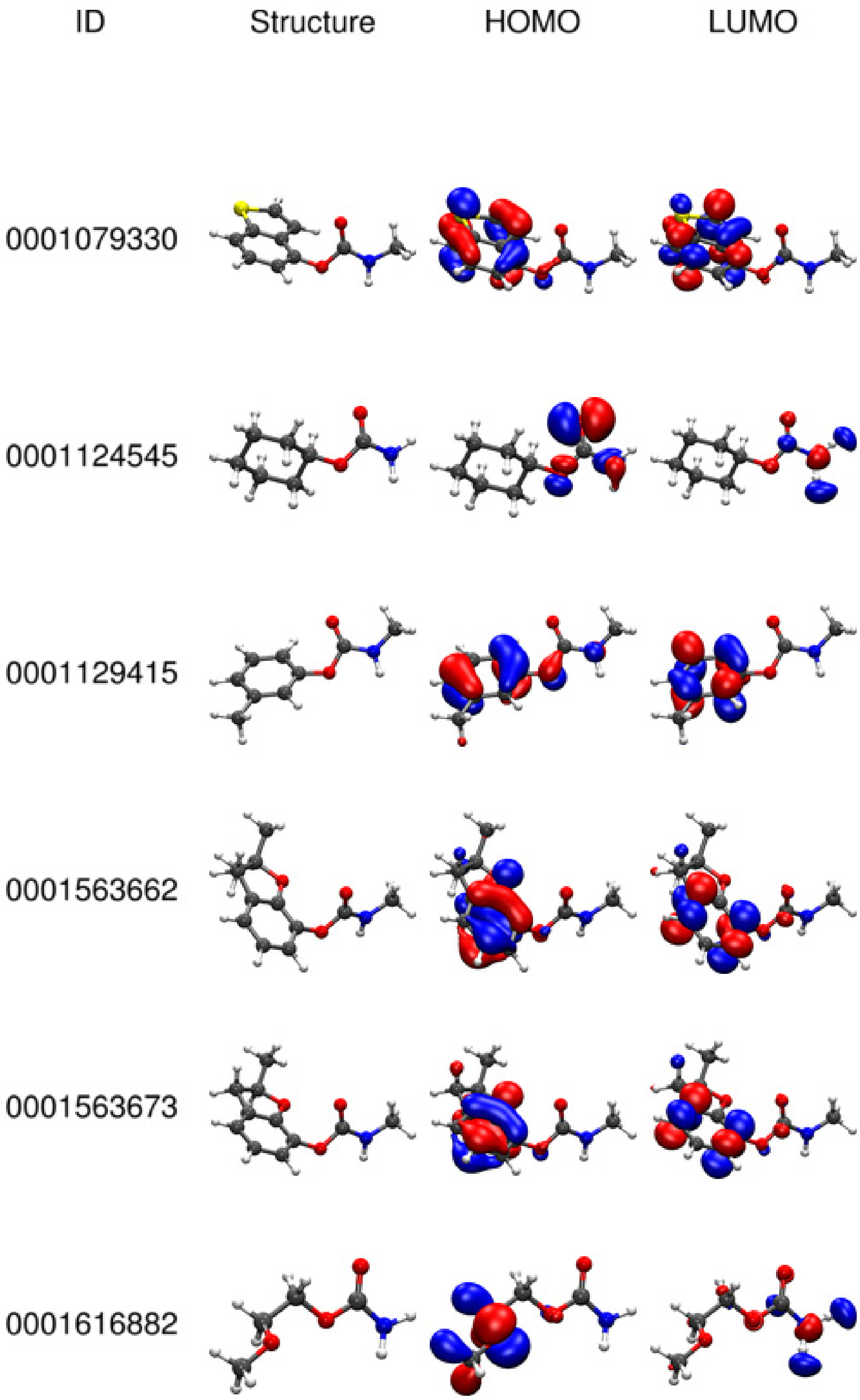

Figure S2. (Cont.)

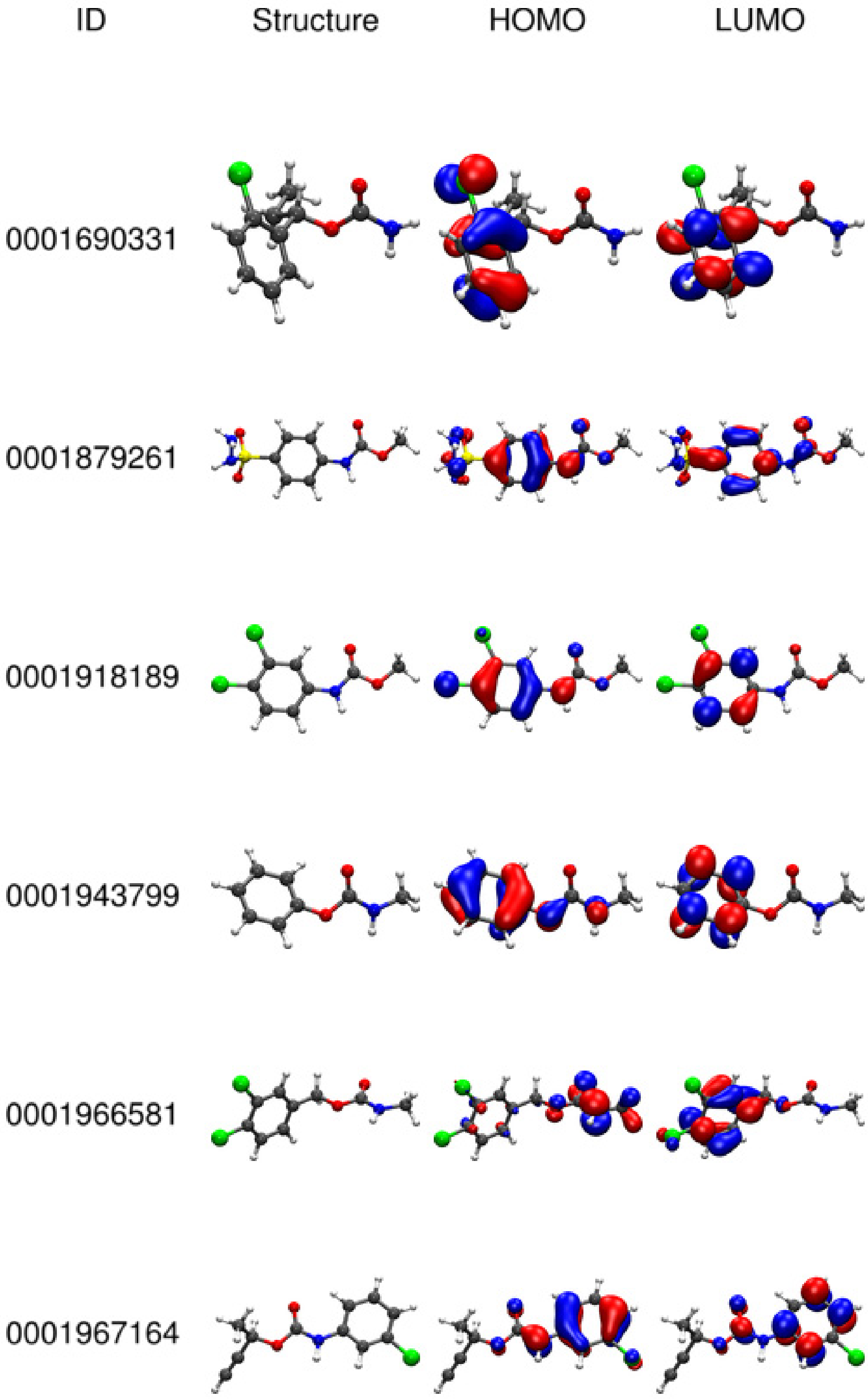

Figure S2. (Cont.)

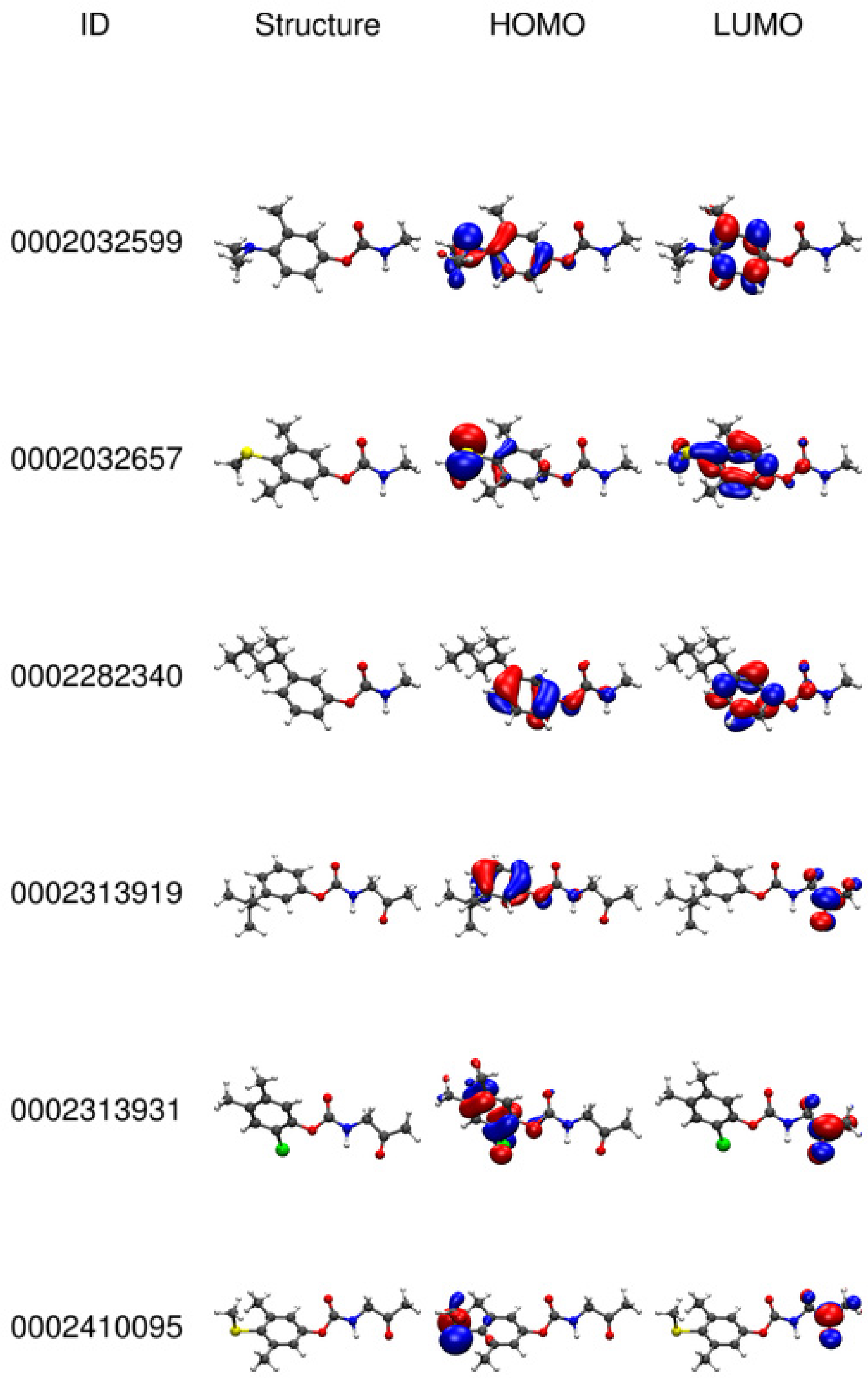

Figure S2. (Cont.)

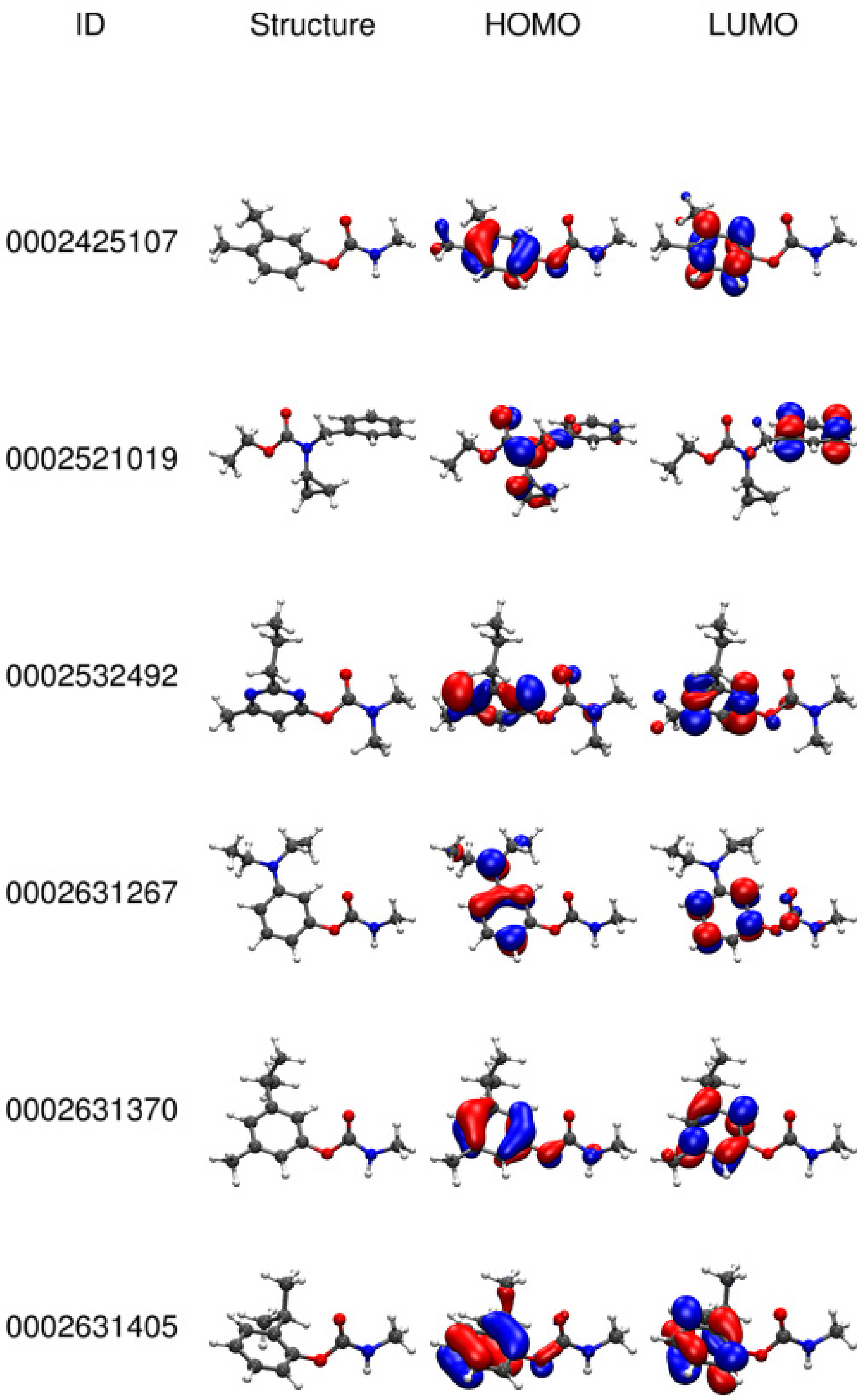

Figure S2. (Cont.)

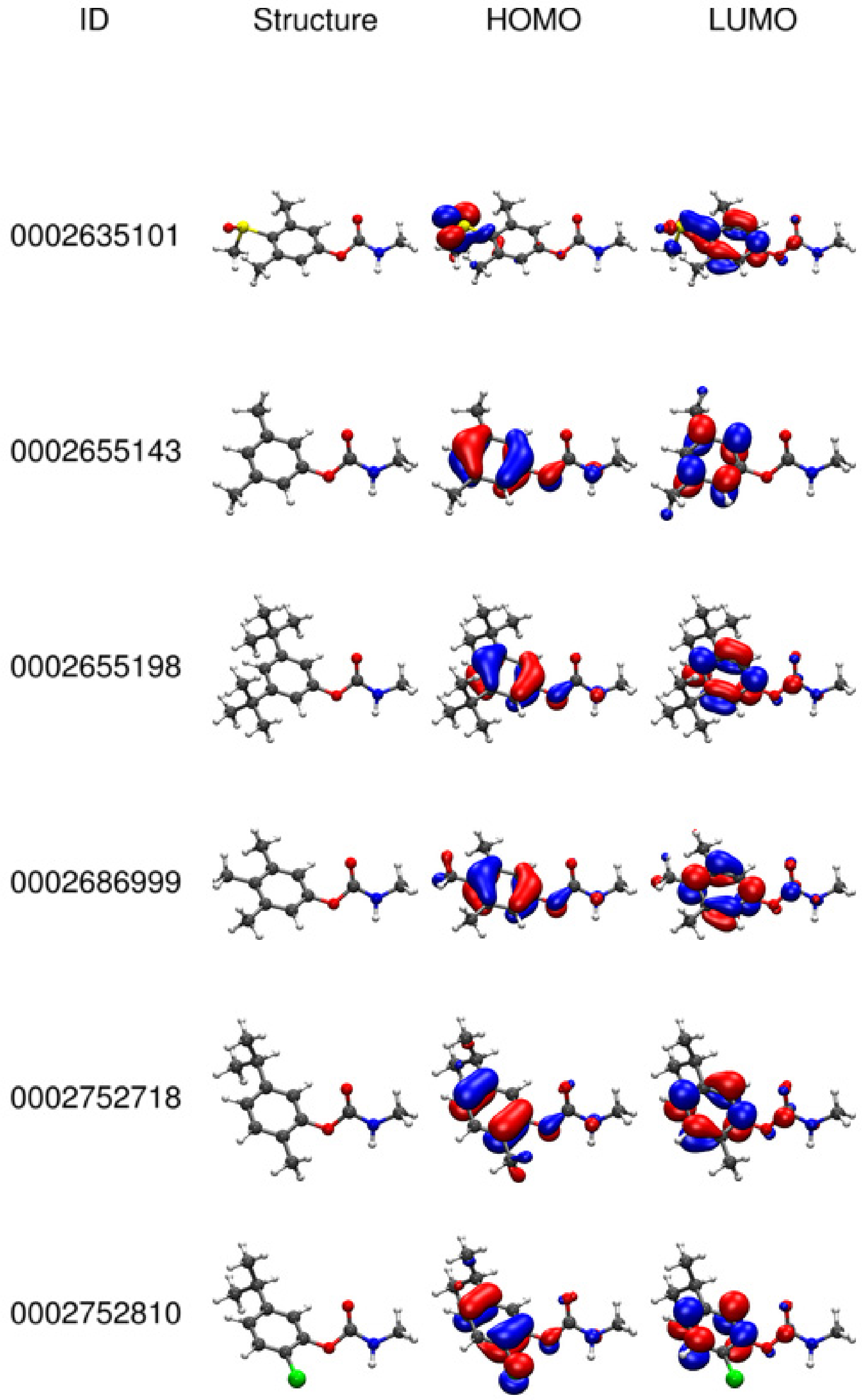

Figure S2. (Cont.)

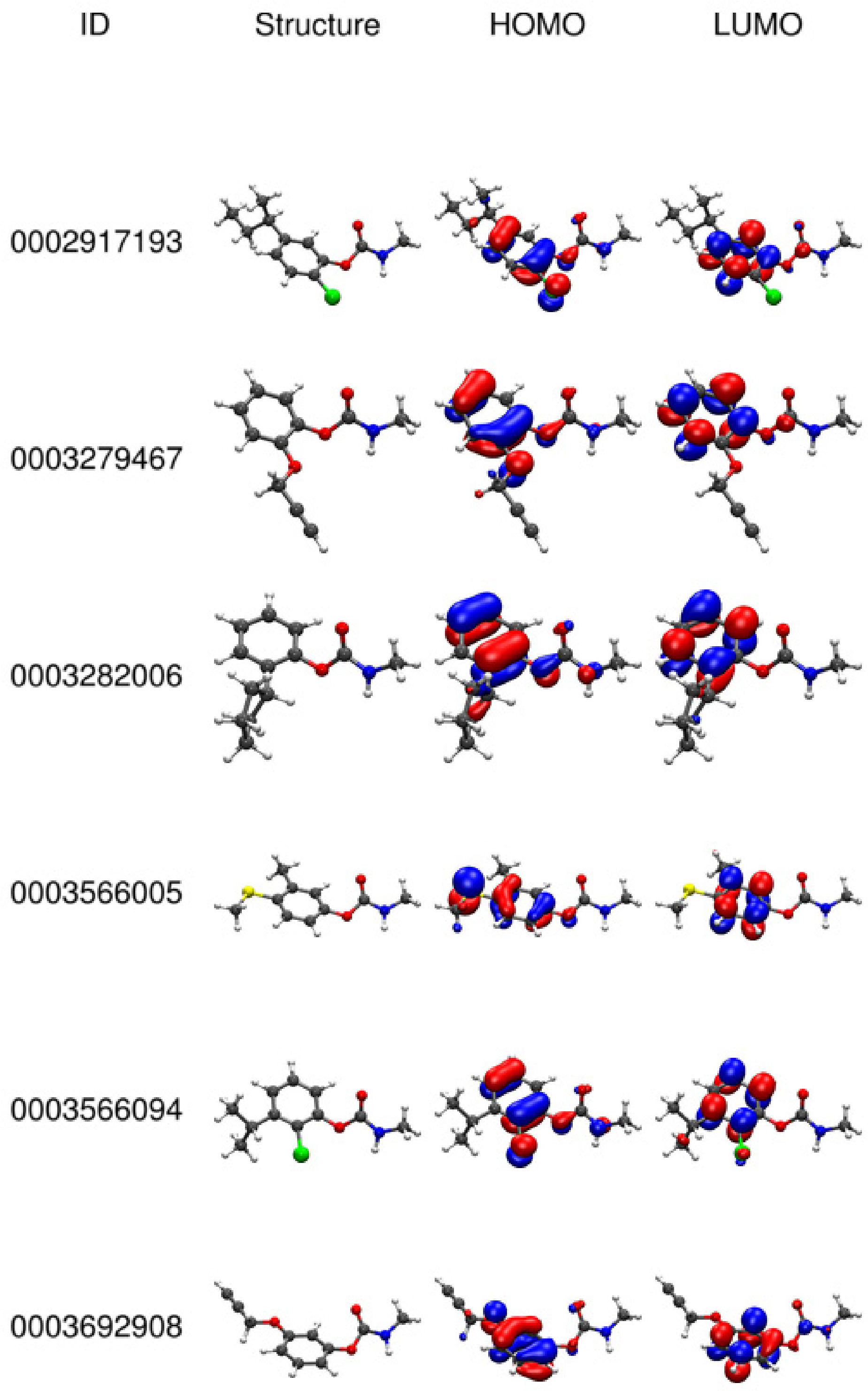

Figure S2. (Cont.)

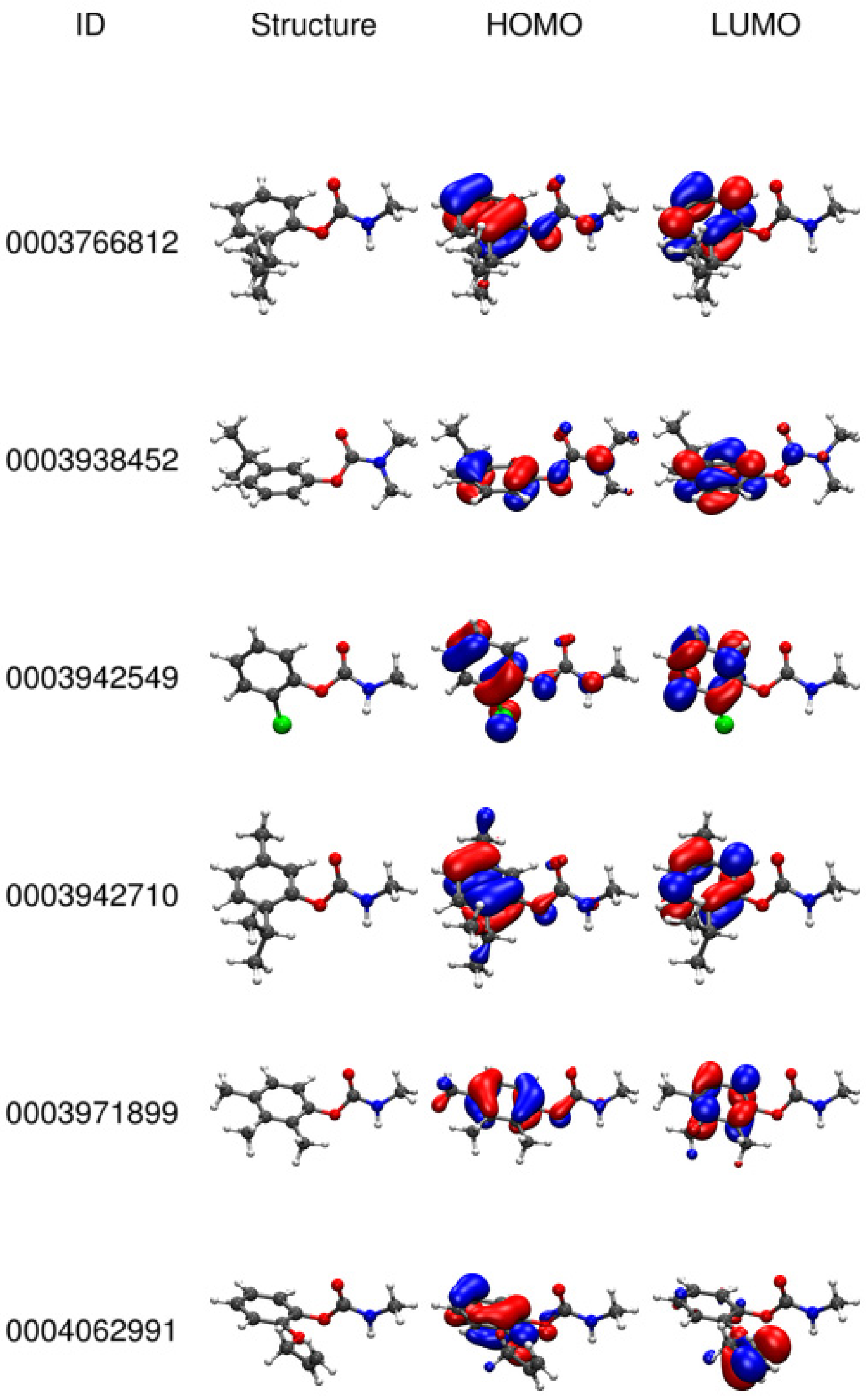

Figure S2. (Cont.)

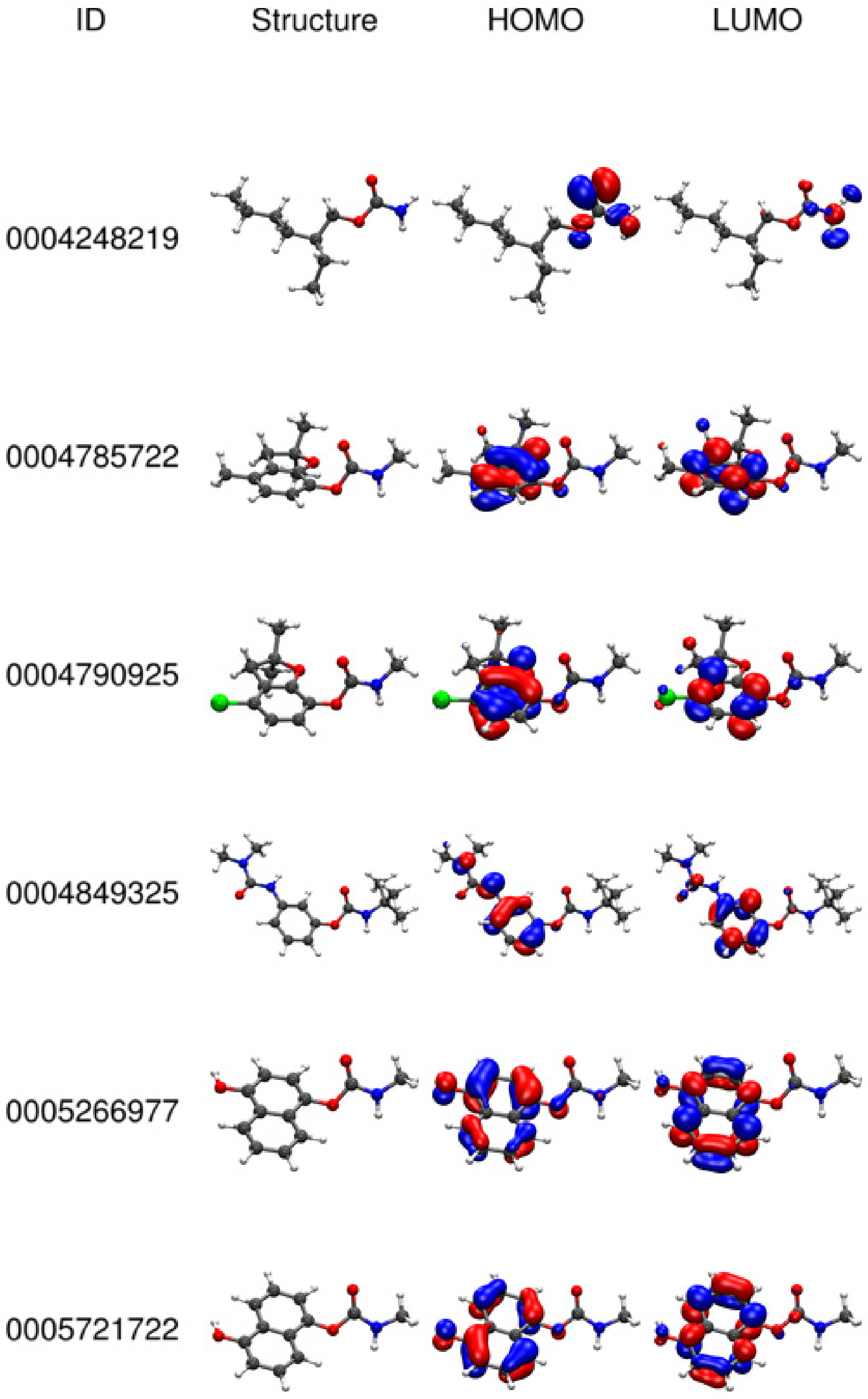

Figure S2. (Cont.)

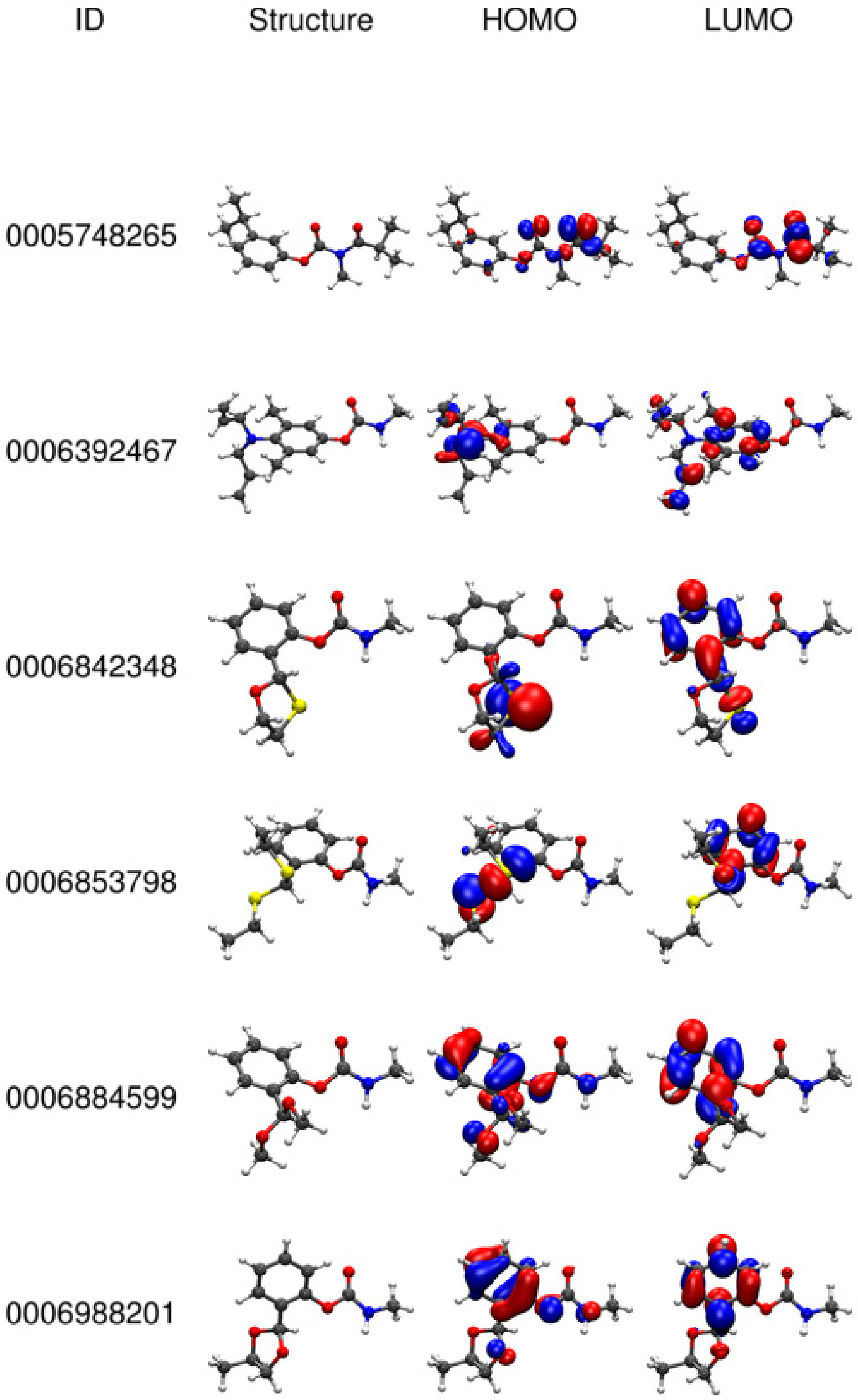

Figure S2. (Cont.)

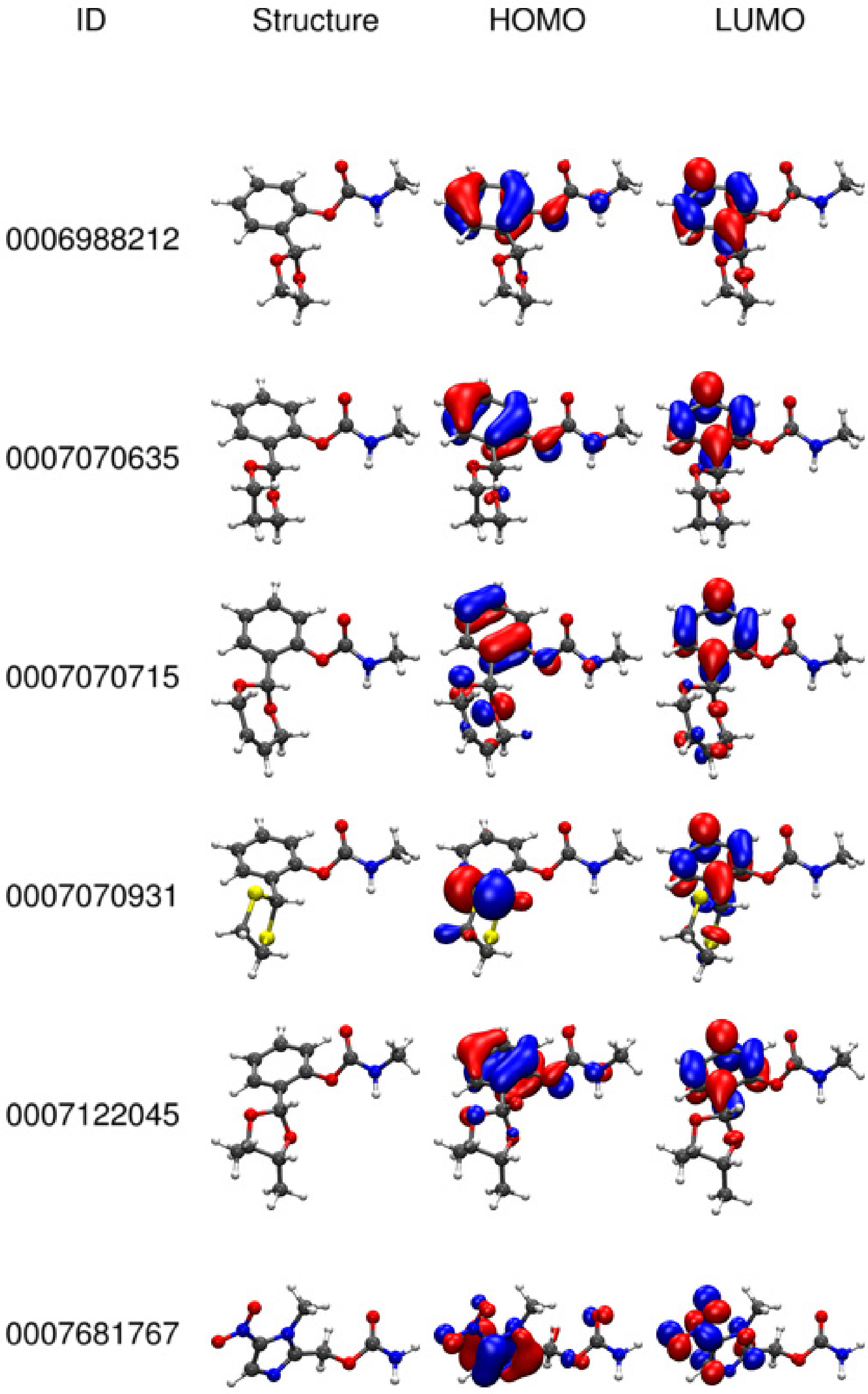

Figure S2. (Cont.)

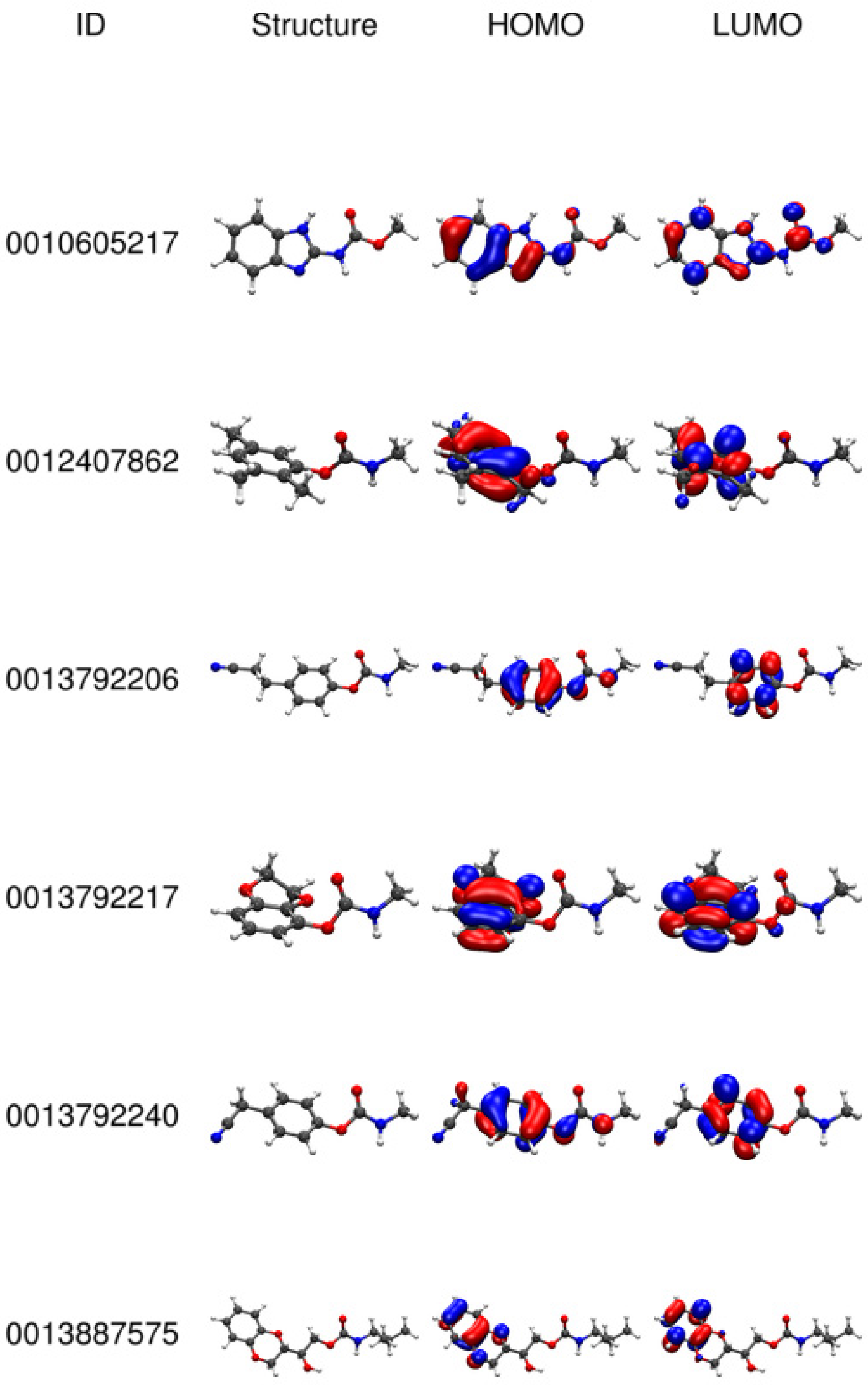

Figure S2. (Cont.)

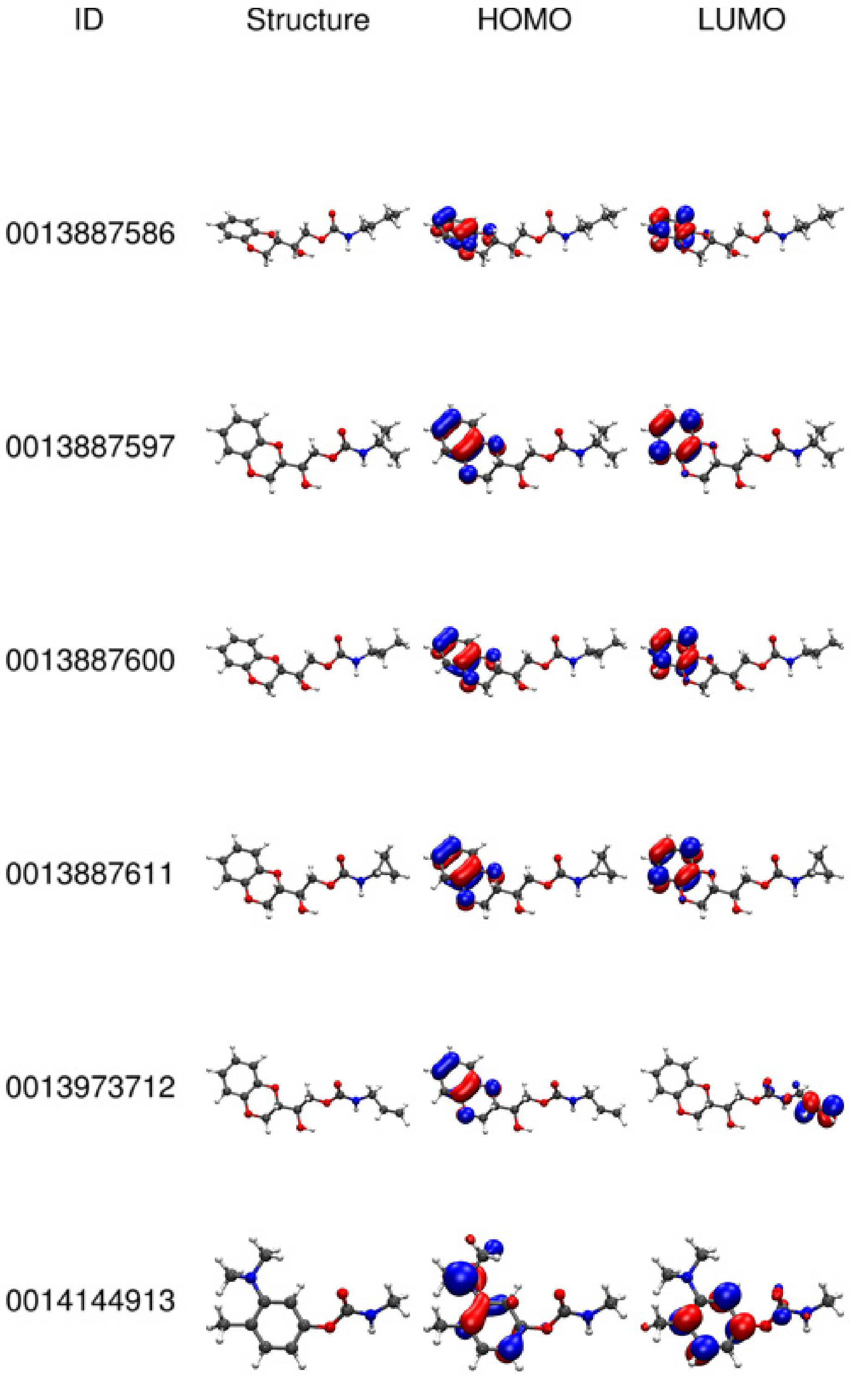

Figure S2. (Cont.)

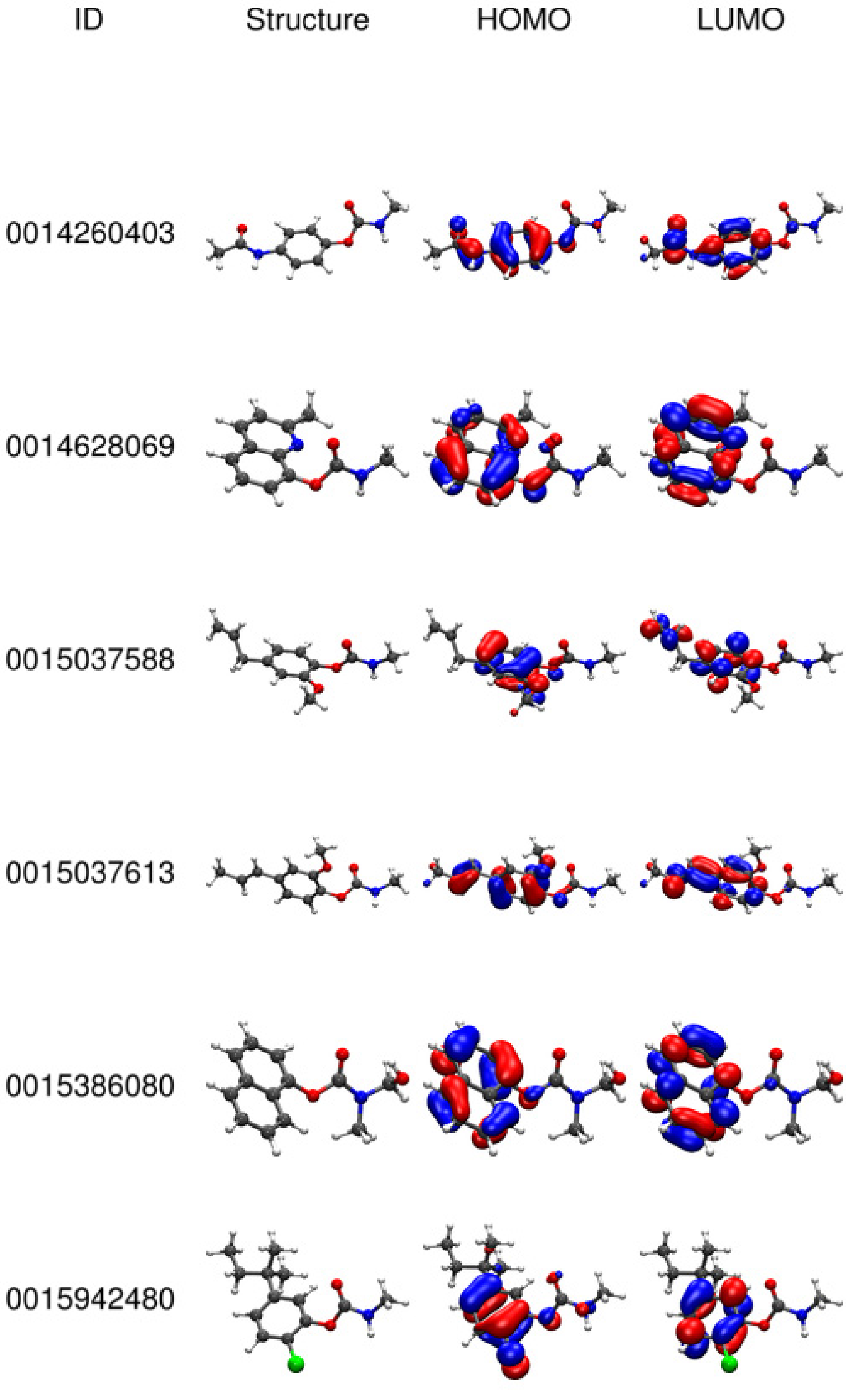

Figure S2. (Cont.)

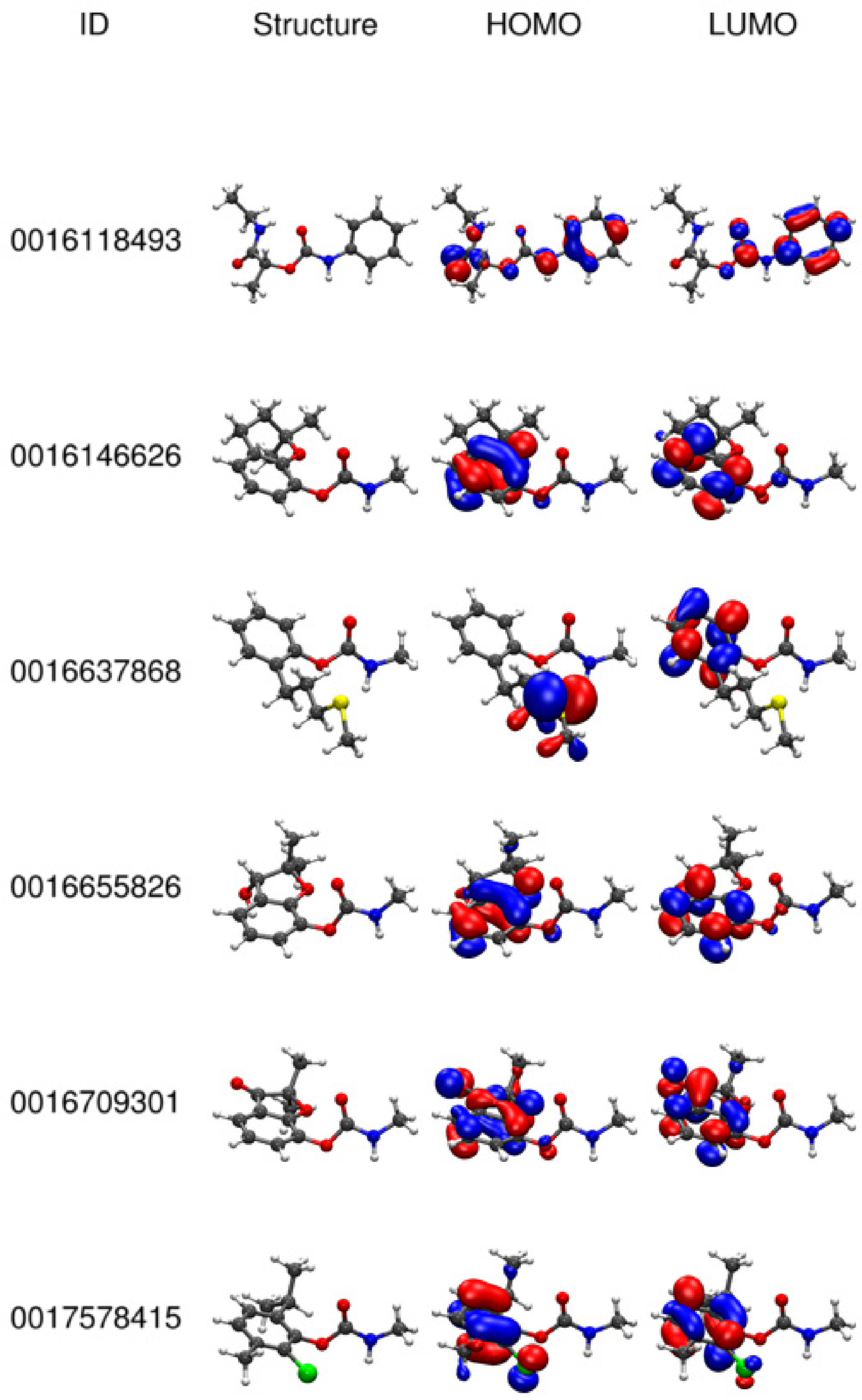

Figure S2. (Cont.)

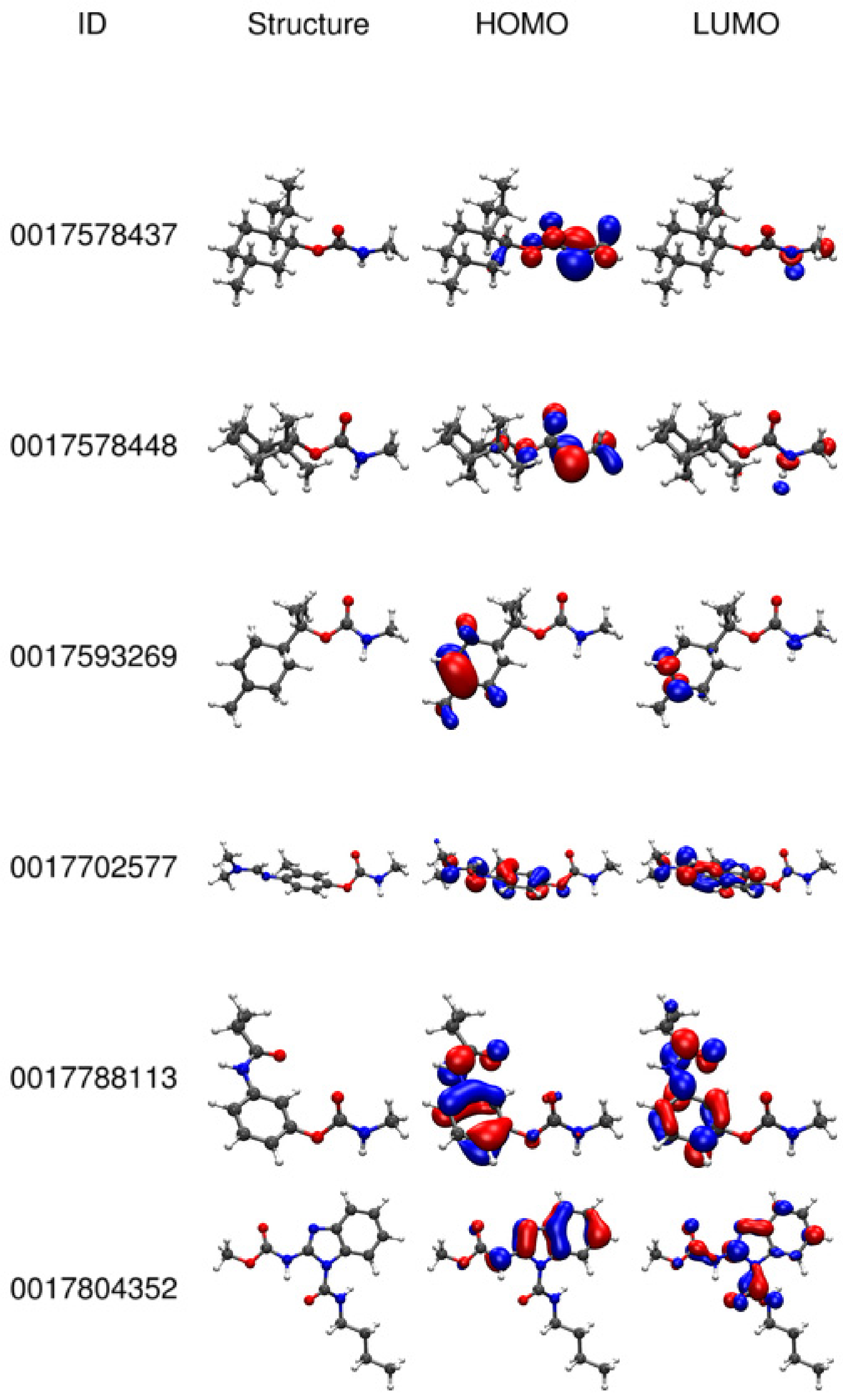

Figure S2. (Cont.)

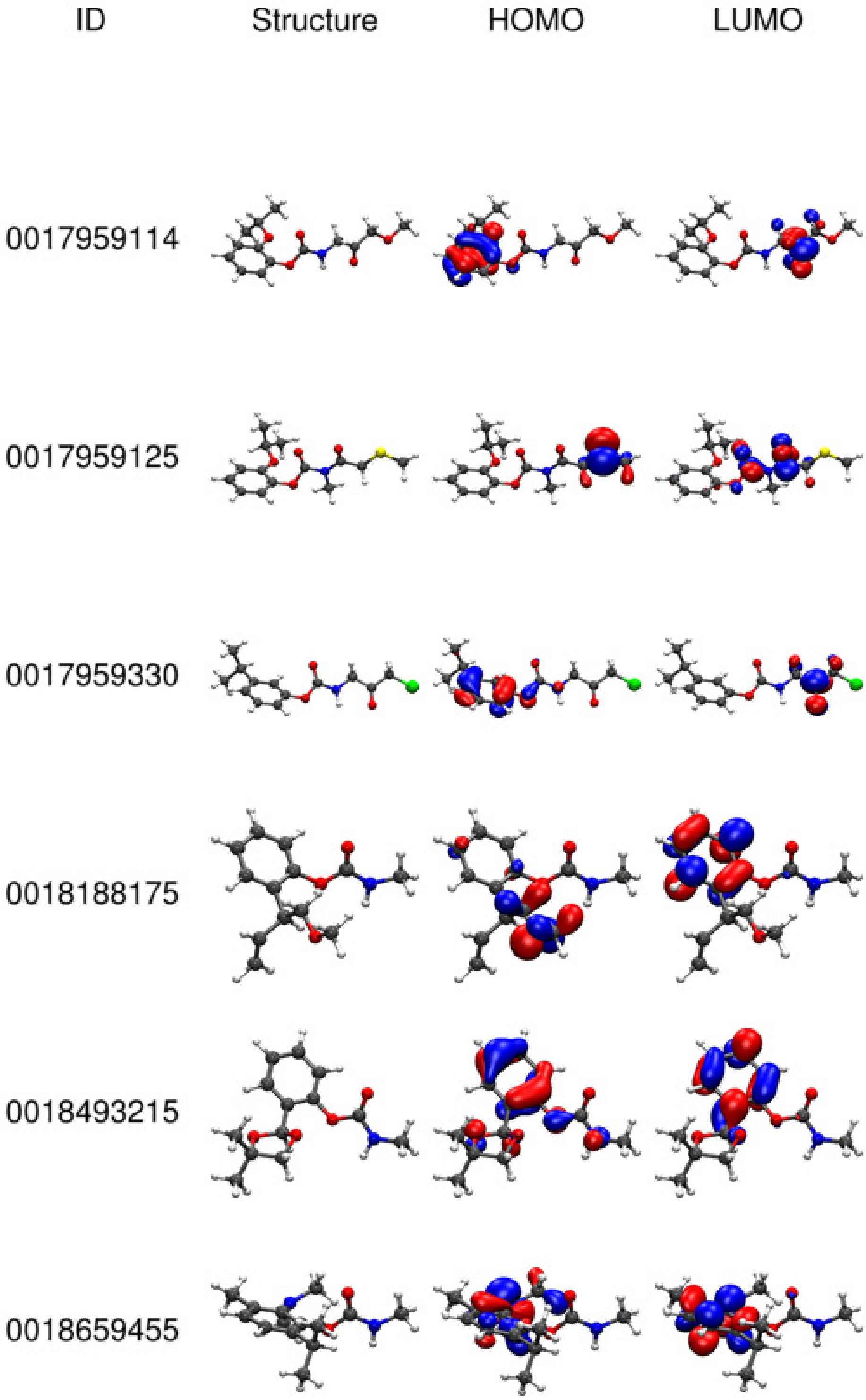

Figure S2. (Cont.)

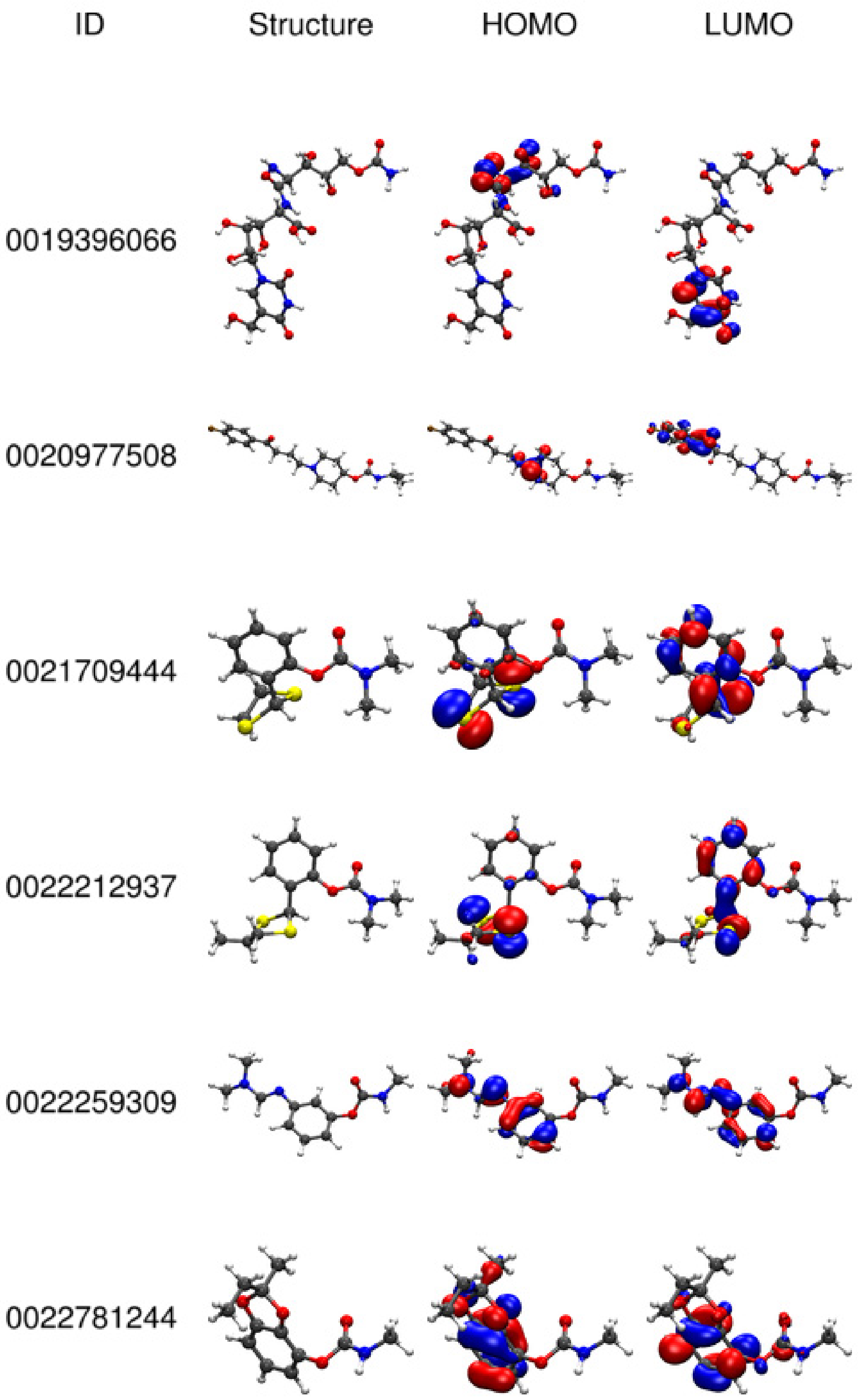

Figure S2. (Cont.)

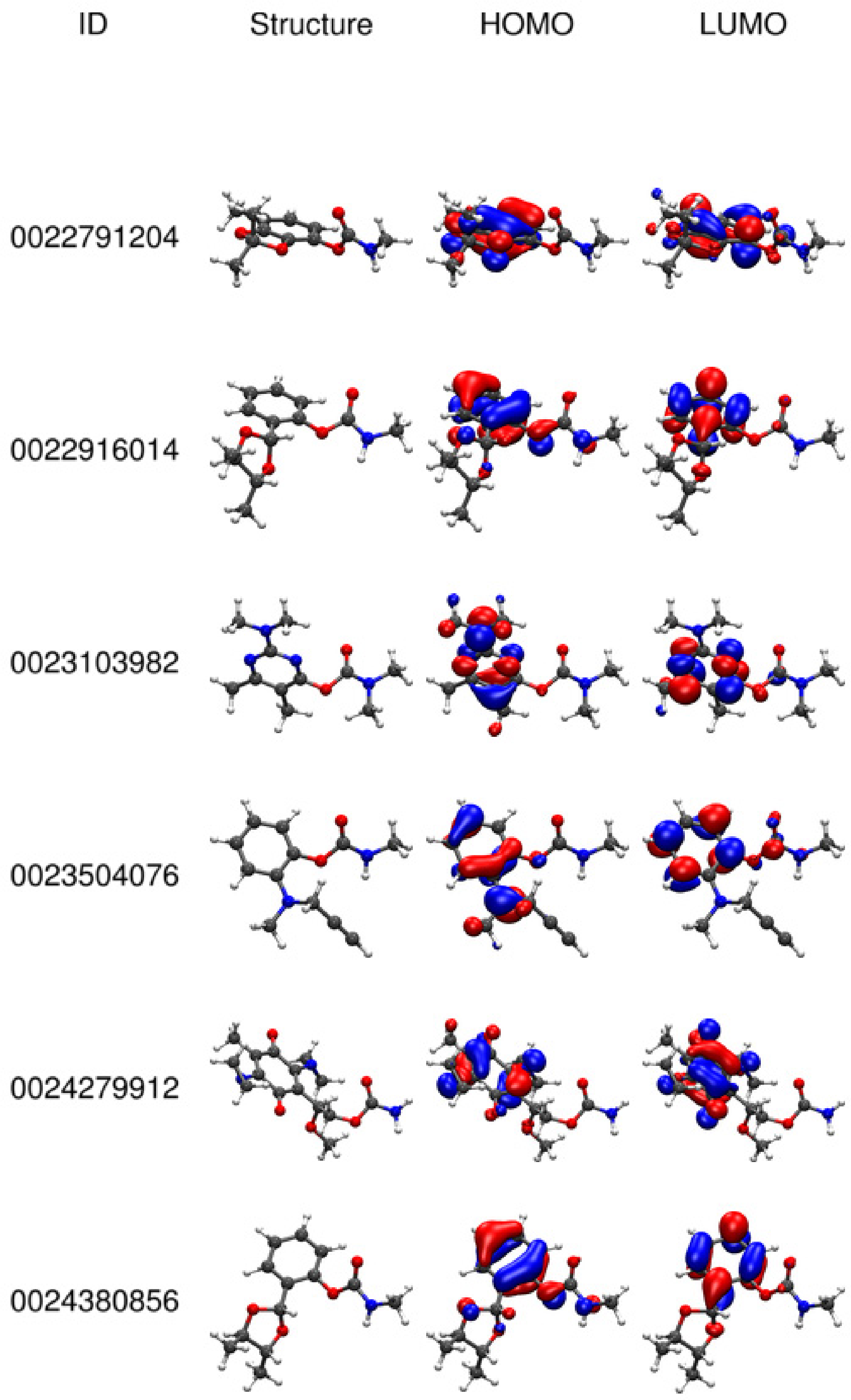

Figure S2. (Cont.)

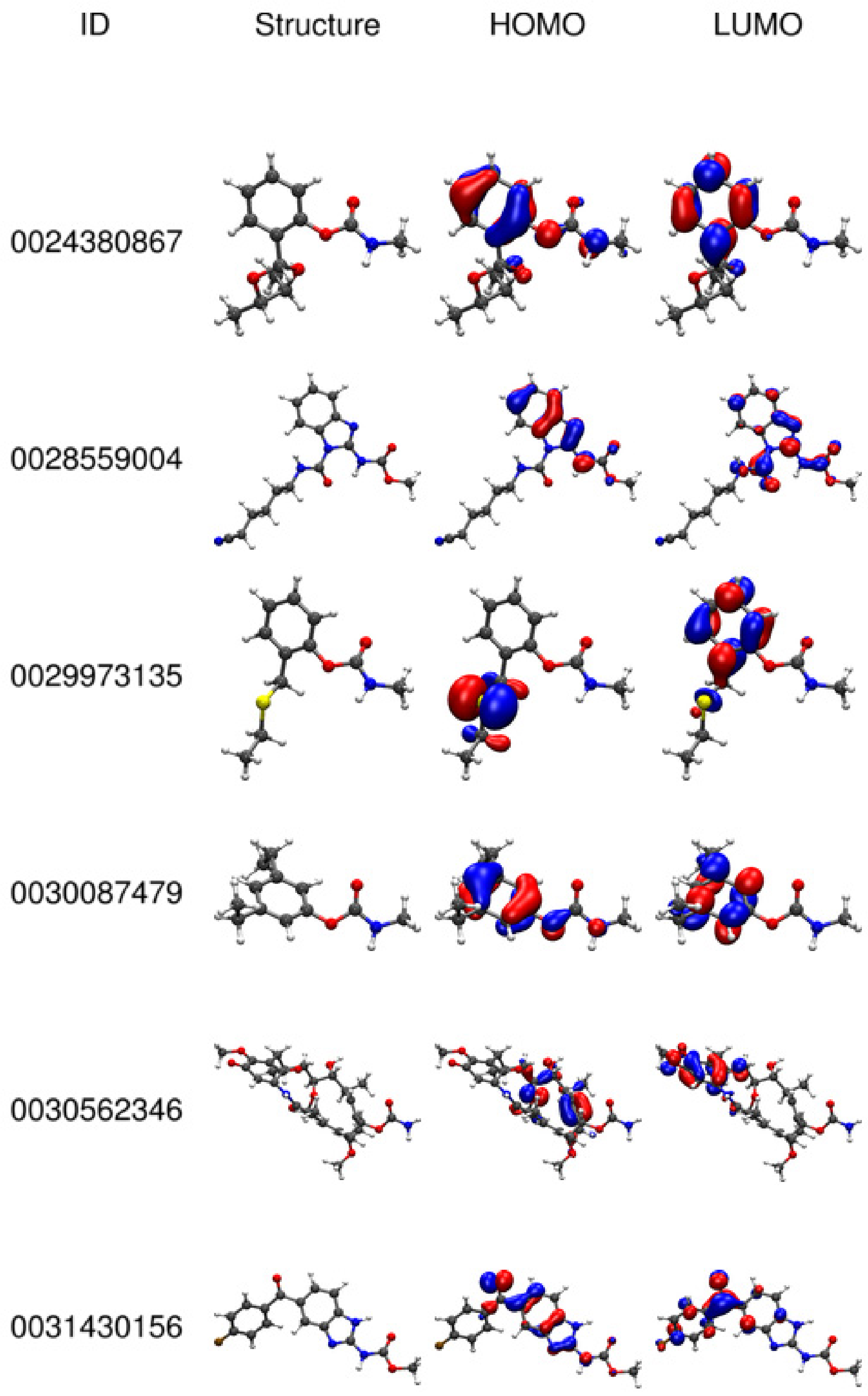

Figure S2. (Cont.)

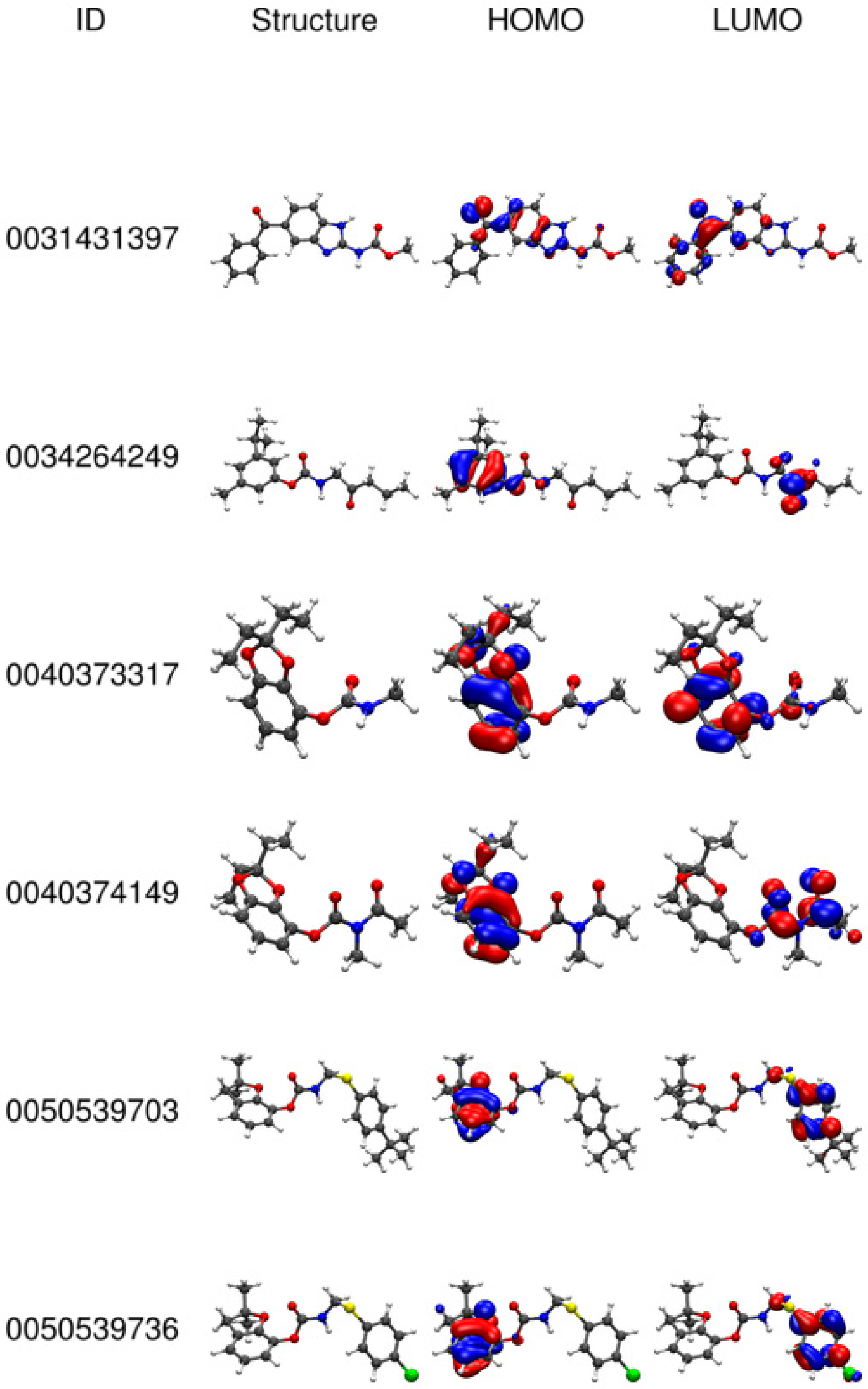

Figure S2. (Cont.)

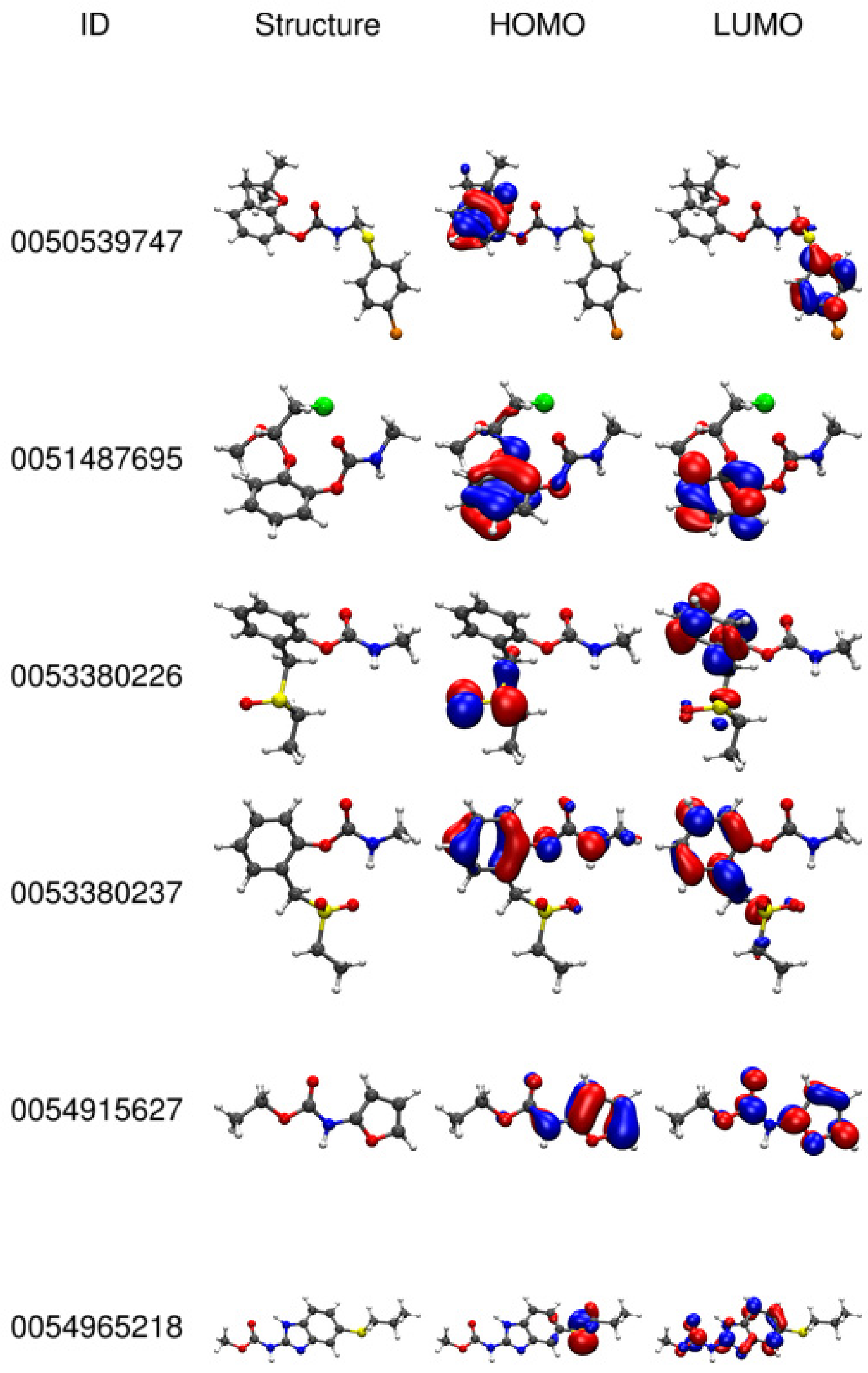

Figure S2. (Cont.)

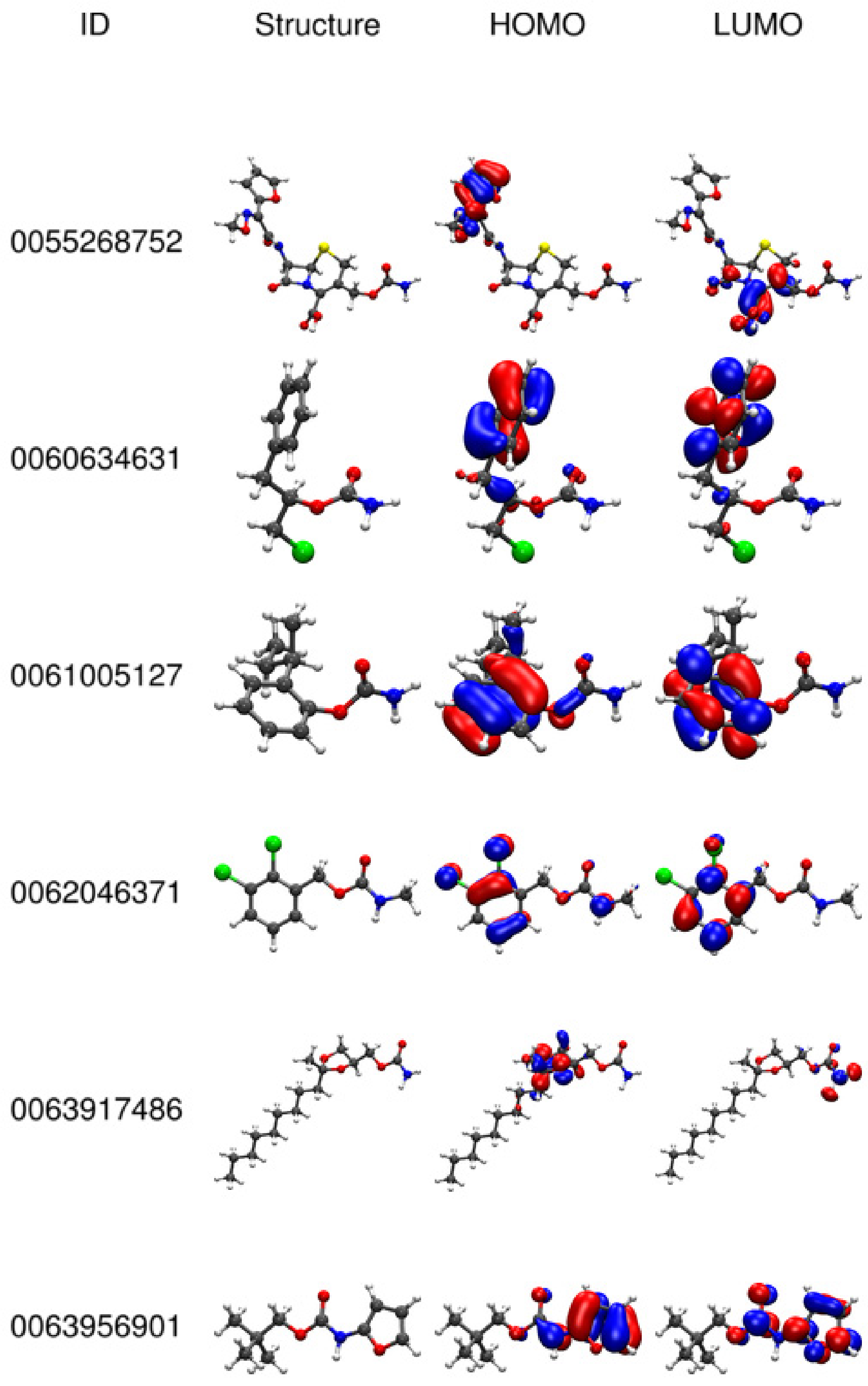

Figure S2. (Cont.)

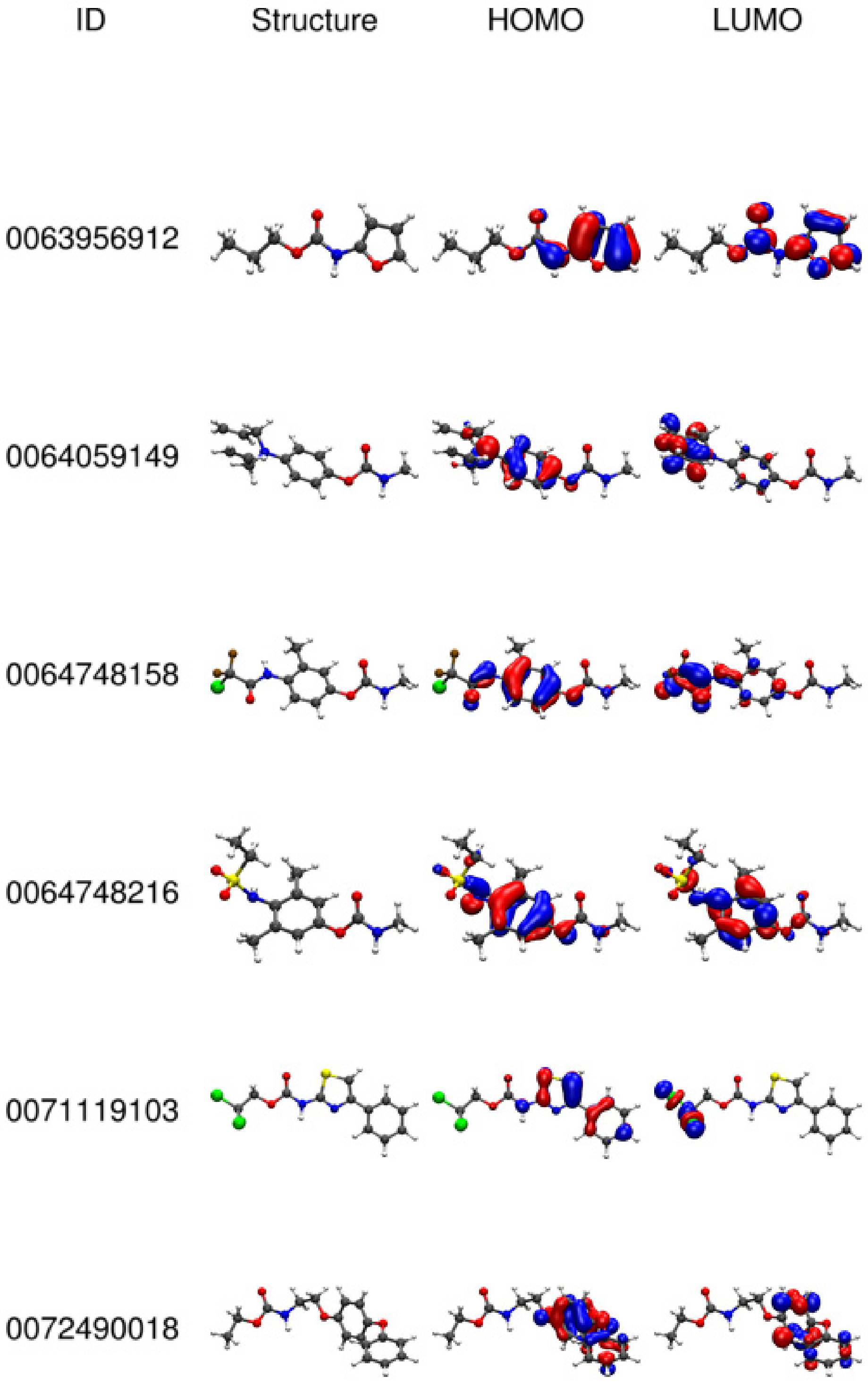

Figure S2. (Cont.)

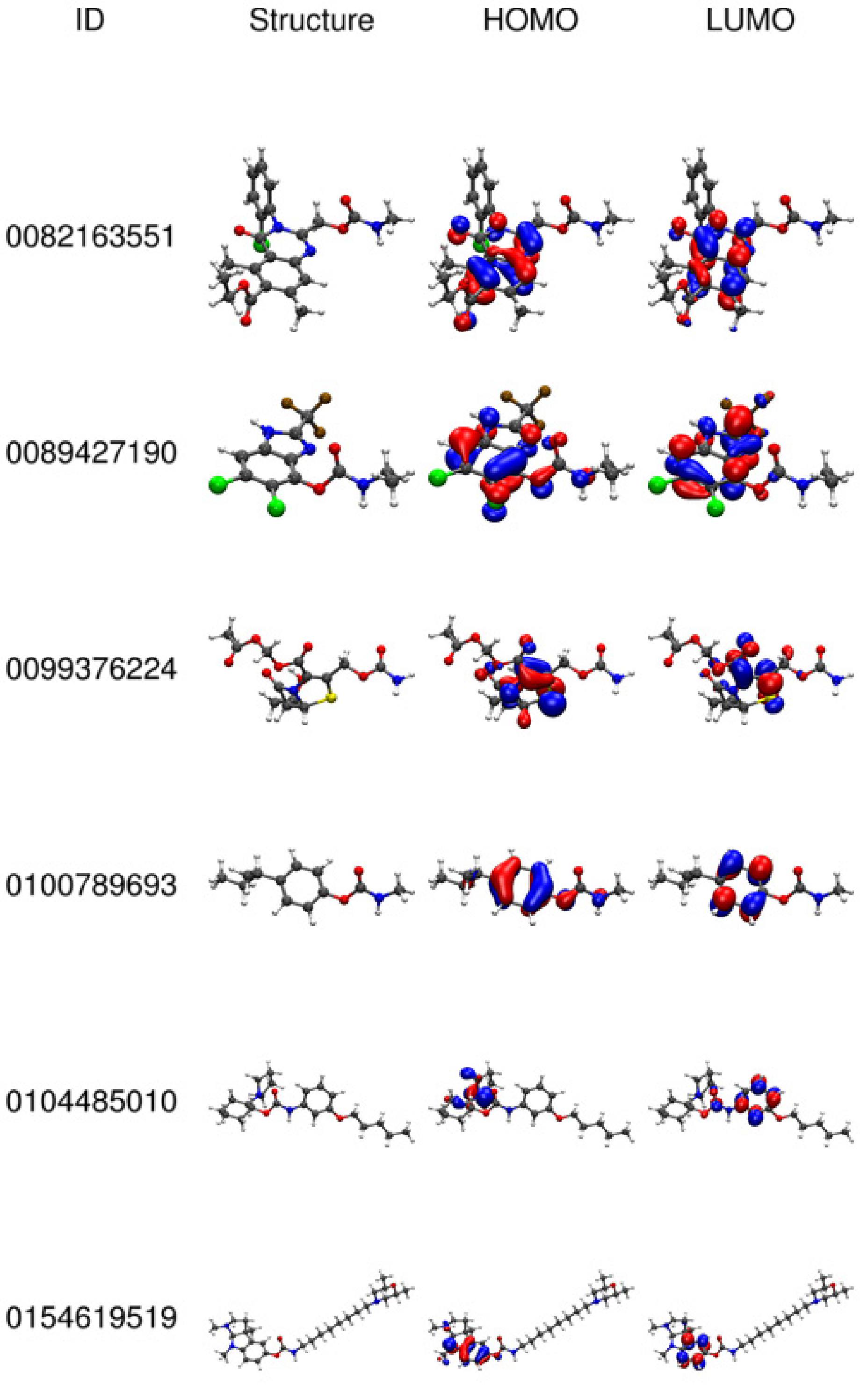

Figure S2. (Cont.)

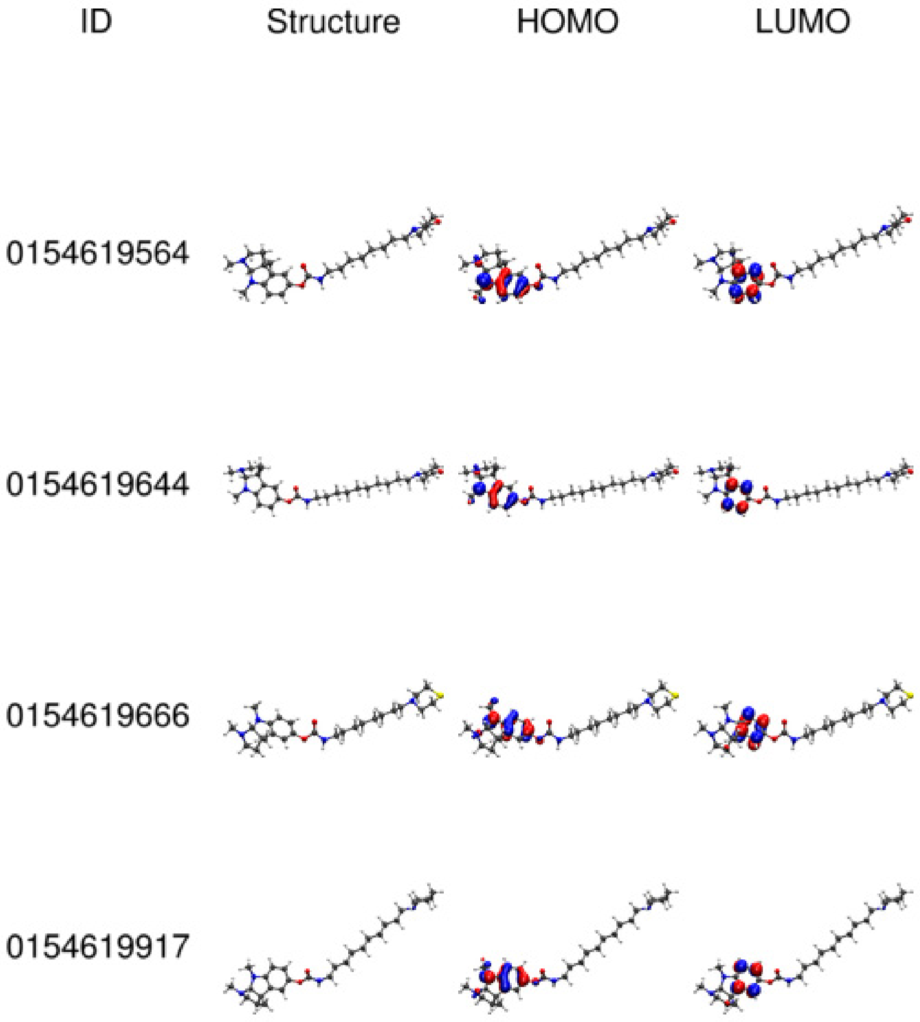

Figure S2. (Cont.)
